# Shifts of the soil microbiome composition induced by plant–plant interactions under increasing cover crop densities and diversities

**DOI:** 10.1038/s41598-023-44104-8

**Published:** 2023-10-10

**Authors:** Derek R. Newberger, Ioannis S. Minas, Daniel K. Manter, Jorge M. Vivanco

**Affiliations:** 1 Department of Horticulture and Landscape Architecture and Center for Rhizosphere Biology, Colorado State University, Fort Collins, CO 80523 USA; 2 Department of Horticulture and Landscape Architecture and Pomology Research, Colorado State University, Fort Collins, CO 80523 USA; 3 USDA, Agricultural Research Services, Soil Management and Sugar Beet Research Unit, Fort Collins, CO 80526 USA

**Keywords:** Plant ecology, Biotic

## Abstract

Interspecific and intraspecific competition and facilitation have been a focus of study in plant-plant interactions, but their influence on plant recruitment of soil microbes is unknown. In this greenhouse microcosm experiment, three cover crops (alfalfa, brassica, and fescue) were grown alone, in paired mixtures, and all together under different densities. For all monoculture trials, total pot biomass increased as density increased. Monoculture plantings of brassica were associated with the bacteria *Azospirillum* spp., fescue with *Ensifer adhaerens*, and alfalfa with both bacterial taxa. In the polycultures of cover crops, for all plant mixtures, total above-ground alfalfa biomass increased with density, and total above ground brassica biomass remained unchanged. For each plant mixture, differential abundances highlighted bacterial taxa which had not been previously identified in monocultures. For instance, mixtures of all three plants showed an increase in abundance of *Planctomyces* sp. SH-PL14 and *Sandaracinus amylolyticus* which were not represented in the monocultures. Facilitation was best supported for the alfalfa-fescue interaction as the total above ground biomass was the highest of any mixture. Additionally, the bulk soil microbiome that correlated with increasing plant densities showed increases in plant growth-promoting rhizobacteria such as *Achromobacter xylosoxidans*, *Stentotrophomona*s spp., and *Azospirillum* sp. In contrast, *Agrobacterium tumefaciens*, a previously known generalist phytopathogen, also increased with alfalfa-fescue plant densities. This could suggest a strategy by which, after facilitation, a plant neighbor could culture a pathogen that could be more detrimental to the other.

## Introduction

The soil hosts interactions between the largest global biomass distribution of terrestrial plants and microbes^1^. Plants shift the biotic environment in the soil to benefit themselves, their offspring, and other plant species^2^. As such, plants and their microbiomes directly or indirectly impact one another through competition and facilitation^3,4^. Understanding the fundamental underpinnings of plant-microbiome feedbacks that manipulate the soil environment would be invaluable for agriculturalists.

Cover cropping is an ancient agricultural technique where plants are grown for the purpose of improving soil health instead of being harvested for profit. Cover cropping can improve acquisition and retention of nutrients in the soil, prevent erosion, and control weeds and pathogens^5–11^. Cover crops have successfully regenerated heavily used agricultural soils^12,13^.

Exemplary cover crops are alfalfa (*Medicago sativa*), which can increase the levels of nitrogen in the soil^14^; mustard plants (*Brassica* sp.), which are known to produce powerful antimicrobials^15^; and grasses (*Festuca* sp.), which prevent erosion, control weeds, and produce large amounts of fibrous roots that sequester organic carbon^5,12,14,16,17^. Non-leguminous cover crops decrease nitrogen leaching and increase soil organic carbon; however, this may promote a yield reduction of the primary cash crop under certain circumstances^12^. Thus, it has been posited that a mix of legume and non-leguminous cover crops is the best method for increasing cash crop yield^12^. However, to effectively combine each plant-specific benefit for agricultural purposes, fundamental knowledge of plant co-existence is vital.

Plant intraspecific and interspecific competition make compatibility and density optimization of cover crops challenging^18,19^. Plant density and diversity are linked to soil microbial community diversity, function, and interaction^20^. Although plant diversity may increase microbial activity and functionality^21^, as different plant species recruit different beneficial microbes, microbe functionality could be distilled due to the lack of compatibility between different plant species.

In this study, the aim was to identify treatments and microbiomes that induce greater total cover crop biomass (not necessarily individual plant biomass), since the increased plant matter provides soil health attributes^17,22^. Furthermore, it was proposed that plant–plant competition leading to a better occupation of space and increased total cover crop biomass could result from enhanced recruitment of beneficial microbes in the soil. Microbial analysis focused on the bulk soil microbiome, which is where the following primary cash crop will be established. This work aimed to use a significant increase of total plant biomass to identify bulk soil bacterial shifts related to plant-plant density or diversity situations.

## Methods

### Soil disinfection and cover crop seed density

Soil was collected from Colorado State University’s Agricultural Research, Development and Education Center South. Large debris were removed from the soil using metal sieves (2 cm wide). Autoclaved soil was used to reduce soil microbial biomass and community complexity and to maximize the impact the plant had on the soil microbiome^23–25^. Soil was homogenized and then autoclaved in batches of approximately 13.5 kg in 61 × 76 cm polyethylene autoclave bags using a STERIS steam autoclave (Mentor, Ohio, USA) for three 40 min liquid cycles at 121 °C. After soils were autoclaved, they were pooled to reduce any potential variability associated with each autoclave cycle. Different seed density maximums were tested prior to the experiment showing 1–3, 24, and 48 plant densities had high seedling survivability, and senescence started at week four.

### Cover crop greenhouse experiment

Plants were grown for 32 days from August 1 to September 1, 2021, in Colorado State University's Horticultural Center Greenhouse Facility. A microcosm was its own “pot” (6 × 4.9 × 5.6 cm) taken from a 36-cell tray, and each microcosm was separated by ~ 2 cm (Supplementary Fig. S1). Pots were lined with a double layer of Vigoro Weed Control Fabric Medium Duty to reduce soil runoff. There were 7 diversity treatments (1. alfalfa, 2. brassica, 3. fescue, 4. alfalfa-brassica, 5. alfalfa-fescue, 6. brassica-fescue, 7. alfalfa-brassica-fescue) and 3 density treatments (low: 1–3 plants, medium: 24 plants, and high: 48 plants) for a total of 21 treatments (Supplementary Table S1). Each treatment had 12 replicates for a total of 252 pots. Random block design of 21 × 12 was configured by an online random block design generator (https://www.randomizer.org). There was one treatment type per column. The reference control for this plant-plant competition/facilitation study was a single cover crop species to exemplify a plant with no competition/facilitation. Cover crop seed mixes and densities were manually counted and placed into microcentrifuge tubes. Each microcentrifuge tube was briefly vortexed to mix the seeds. Seeds were spread evenly into the pots with autoclaved soil using tweezers, which were washed with ethyl alcohol in between samples. To remedy seed germination failure, pregerminated seeds were planted into each pot 7 days into the experiment to reach the target densities. Plants were watered daily at water holding capacity with DI water to reduce the introduction of microbes and other contaminants. Additionally, DI water was used to mimic uncontaminated rainwater since cover crops are ideally not irrigated. As an aside, seeds were not sterilized as to prevent additional seed death and to maintain fundamental microbes on the surface of the seed coat for the respective plant. At the end of the experiment, the number of plants in each pot were counted.

### Bulk soil collection

Bulk soil samples were collected at the end of the study, and prior to biomass harvest. Bulk soil refers to the surrounding soil, which has been influenced by an organism such as a plant but excludes the soil adhering to the roots which is known as the rhizosphere^26^. Within each treatment, the top five replicates that best represented target densities were selected for bacteriome analysis. A core borer (1.5 cm diameter) was used to collect the surrounding bulk soil from the center of the pot without disturbing the above-ground biomass. The soil probe was sterilized between samples. Visible soil debris was scrubbed off the soil probe using a brush soaked in a tap water-Alconox (White Plains, New York, USA) solution. Next, the soil probe was rinsed with 2% bleach followed by 70% ethyl alcohol. Bulk soil cores were placed in a 15 ml falcon tube and immediately stored at − 20 °C. Bulk soil samples were taken over four days.

### Plant biomass

Above ground biomass was measured for every sample (n = 235) and was harvested using scissors, which were surface sterilized between samples using a Bacti-Cinerator III (Monoject Scientific, St. Louis, Missouri. 63103, USA). For each pot, above ground biomass was separated from below ground biomass. If there was more than one plant was growing in the pot, then the above ground biomass was also separated by crop type as well. Plant biomass was oven dried for 72 + hours, and then weighed.

### DNA extraction

Closely following Qiagen’s protocol, total genomic DNA (gDNA) was extracted from 0.25 g of surrounding bulk soil in a Qiagen QIAcube instrument (Germantown, Maryland, USA) using Qiagen PowerSoil Pro ® DNA kits. Any roots and their respective adhering soil were removed from the bulk soil that was to be used for DNA extraction. Elution volume for extractions was of 100 μl. An Invitrogen Qubit fluorometer (Waltham, Massachusetts, USA) quantified DNA concentrations with high sensitivity assay solutions. Bulk soil samples (n = 103) taken from each of the 21 treatments had 4–5 replicates that were randomly selected for DNA extraction. Controls used were pre-extracted Zymo gDNA (Zymo Research Corporation, California, USA) (n = 2), extracted HPLC water (n = 2), PCR 2 HPLC water (n = 2), and pre-extracted and sequenced soil (n = 2).

### Oxford nanopore library prep, sequencing, and bioinformatics pipeline

Extracted DNA was diluted 5 times with HPLC water based on Qubit concentrations (ng/μl). Bacterial primers used were Bact_27F-Mn (5′ –TTTCTGTTGGTGCTGATATTGCAGRGTTYGATYMTGGCTCAG—3′) and Bact_1492R-Mn (5′ACTTGCCTGTCGCTCTATC TTC TACCTTGTTACGACTT—3′). Polymerase chain reaction (PCR) settings were 98 °C for 30 s, 98 °C for 15 s, 50 °C for 15 s, and 72 °C for 1 min for 25 cycles, and 72 °C for 5 min. After the first PCR, equal volumes of DNA and beads were mixed. A 96-pronged magnetic stand was used to move beads with adhering DNA into two 30 s rinses of 70% ethanol. DNA was eluted in a 96-well plate with 40 µL PCR grade water, and beads were removed using a magnetic stand. DNA was quantified using a Qubit with high sensitivity assay solutions. The second PCR settings were 98 °C for 30 s, 98 °C for 15 s, 62 °C for 15 s, and 72 °C for 1 min for 25 cycles, and 72 °C for 5 min. After the second PCR, DNA and barcodes (EXP-PBC-96) were pooled in AMPure bead solution in a 96-well plate. Wells with suspended DNA and barcodes were pooled into a clean Lo-Bind tube. MinION sequencer was loaded with a flow cell (R9.4.1). To prepare the flow cell, air (~ 20 µL) was removed using a pipette. The flow cell was then primed with flush buffer, and pooled DNA was loaded into the sampling port. MinKNOW software was used to sequence the pooled library for 48 h. Raw data was base-called and demultiplexed using Guppy v6.0.1 and reads were then filtered by quality (Filtlong minimum length: 1000; mean quality: 70) and length (Cutadapt: -m 1000 -M 2000). Bacterial taxa were identified using EMU NCBI Reference Database. Sequencing data was processed using DADA2 which removed all singletons by default. EMU error correction removed identified bacterial taxa based on alignment and abundance profiles, such that bacterial taxa with < 1 per 10,000 reads were removed. Sequencing data came from three separate sequence runs, which were pooled for data analysis.

### Statistical analysis

Statistical analyses were run, and figures were made using RStudio Version 1.4.1103. Rarefaction curves show that samples plateaued (Supplementary Fig. S2). Normality for the biomass was tested using the Shapiro–Wilk normality test for normality. Linear models of residuals were used to assess the equality of variance. One-way analysis of variance followed by the Tukey HSD test were used to denote the compact letter display to indicate significance using emmeans, multcompView, and dplyr packages^27–29^. PERMANOVA was used to find significant differences between treatments and visualized with a constrained Principal Coordinate Analysis (PCoA) with Bray–Curtis dissimilarity index used as a distance from the Vegan package^30^. Betadisper from the Vegan package was used to measure the homogeneity of multivariate dispersions. Differential abundance analysis was based on bacterial species counts using log2 fold change with the Benjamini–Hochberg method^31^ using the fdr (false discovery rate) function at an adjusted p-value threshold of 0.05. Alpha diversity was visualized using the Shannon diversity index through the phyloseq package using rarified data (M = 31,960, SD = 11,508, reads per sample)^32^.

### Rights and permissions for research involving plants

This study did not require special right or permissions for plant material use. Seeds (fescue mixture from Vitality, ranger alfalfa and Mighty Mustard® Pacific Gold from Johnny’s selected seeds) used in this greenhouse study were not from wild plants and were purchased and are not listed as an endangered species.

## Results

### Monoculture

#### Monoculture plant biomass

In monoculture, total biomass of all three cover crops increased with crop density. Total biomass of alfalfa increased significantly within each density increment and had the highest biomass of any cover crop for densities of 24 plants and 48 plants (Supplementary Fig. S3). Brassica above ground biomass did not statistically differ between 1 and 24 plants but required a density of 48 plants to raise the total biomass. Fescue biomass at a density of 24 plants was double the same at a density of 1 plant; the biomass was not significantly different between 24 and 48 plants. For all three cover crop types, a single plant density yielded the largest individual plant, and the number of plants-to-biomass ratio was inversely proportional to density.

#### Surrounding bulk soil bacteriome analysis

**Figure 1:**
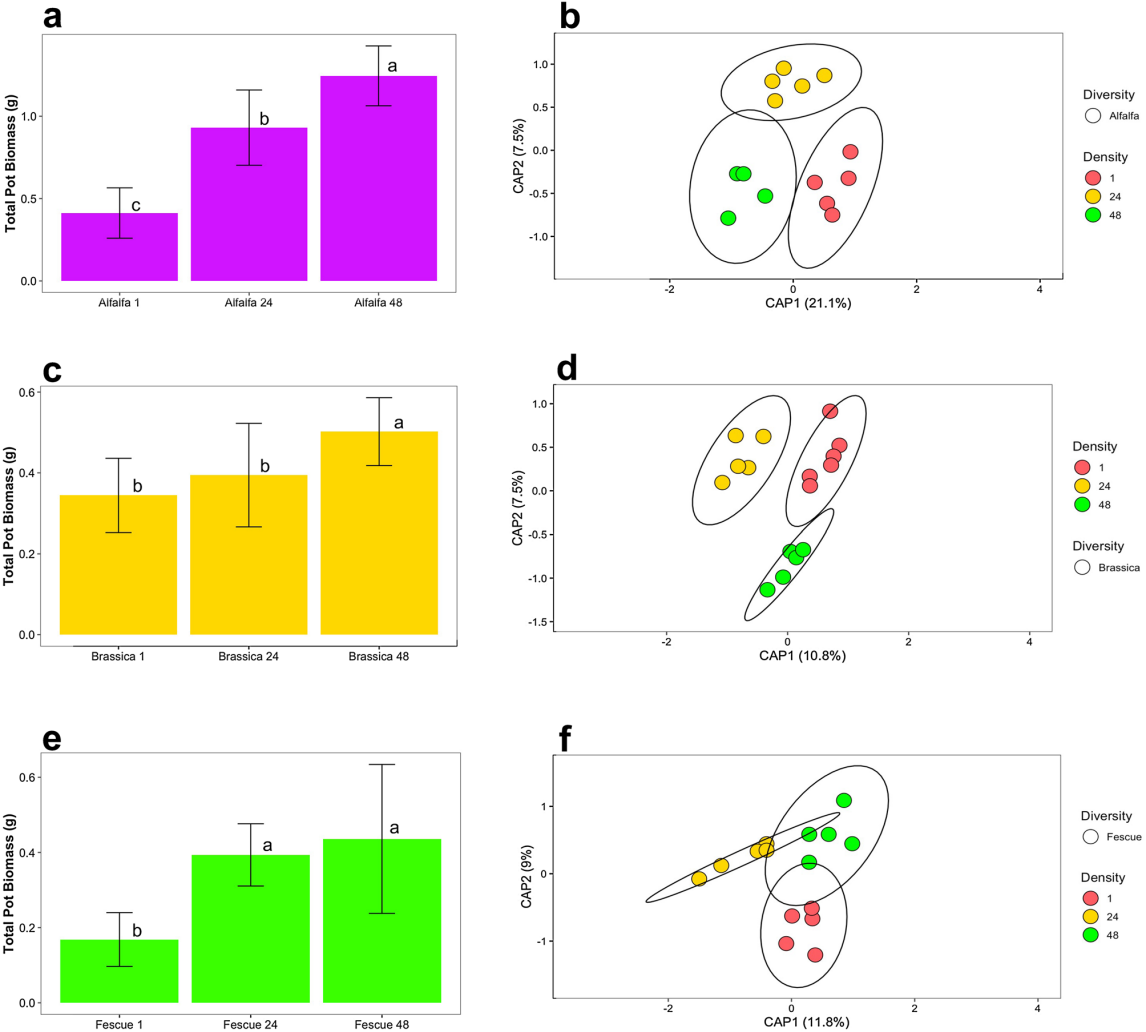
Above ground dry biomass in monoculture for crop densities of one plant total, 24 plants total, and 48 plants total. (**a**) Alfalfa dry biomass (purple), (**c**) brassica dry biomass (gold), and (**e**) fescue dry biomass (green). Constrained Principal Coordinate Analysis (PCoA) using Bray–Curtis distance for comparing bulk soil bacteriomes of increasing crop densities by each individual crop (**b**) alfalfa, (**d**) brassica, and (**f**) fescue. Colors were used to represent increasing densities from 1 plant (red), 24 plants (yellow), and 48 plants (green). Letters (a, b, and c) indicate significant differences between the mean values of plant biomass with (Tukey *P* < 0.05). Error bars are the SD.

Bacterial microbiome shifts in the surrounding bulk soil of the plants were assessed using alpha and beta diversities, and differential abundance of specific taxa. For Shannon Diversity Index, there was no consistent visual trend of an increasing alpha diversity measure by density or diversity (Supplementary Fig. S4). PERMANOVA model with all data combined showed both density and diversity were significant factors along with their interaction (Supplementary Fig. S5). Low to high plant densities of alfalfa induced significant (*p* = 0.001, R^2^ = 0.286) shifts on the surrounding bulk soil bacteriome (Fig. 1b, Supplementary Table S2). As an estimate of beta diversity, the average distance to the median for the bacterial bacteriomes at a density of a single alfalfa plant was 0.2893, for 24 alfalfa plants 0.3251, and for 48 alfalfa plants 0.2841. The density of 48 alfalfa plants had the highest clustering out of the three densities. Increasing densities of brassica induced a significant (*p* = 0.01, R^2^ = 0.183) shift on the surrounding bulk soil bacteriome (Fig. 1d, Supplementary Table S2). For brassica, average distance to the median for the bacteriomes had the highest clustering for the single (0.2861), 24 (0.3165), and 48 (0.3047) plant densities. The PERMANOVA test showed that when looking at fescue by increasing densities of 1, 24, and 48 plants, the shift induced on the soil bacteriome was significant (*p* = 0.002, R^2^ = 0.208) (Fig. 1f, Supplementary Table S2). For fescue, the average distance to the median for the bacteriome had the highest clustering for the single plant density with 0.2862, with 24 fescue plants at 0.3227, and 48 fescue plants at 0.3367. The increasing density of the alfalfa monocrop explained the highest variability (CAP1 + CAP2: 28.6) as compared to brassica (18.3) and fescue (20.8) (Fig. 1b,d,f).

Differential abundance comparisons of the bacteriome in the surrounding bulk soil were conducted when there was a significant difference in total plant biomass per pot as density increased (Table 1). Bacteria of interest were those which were highlighted by the differential abundance comparison in conjunction with an increase in total plant biomass. Alfalfa with a low density (one plant) was enriched for 22 bacterial taxa compared to medium (24 plants) and high density (48 plants) microcosms. Alfalfa with a medium density was enriched for 9 bacterial taxa compared with high density microcosms. Alfalfa with a high density was enriched for 13 bacterial taxa compared to both medium and low-density microcosms. Brassica with a low density (one plant) was enriched for 7 bacterial taxa compared to high density (48 plants) microcosms. Brassica with a medium density (24 plants) was enriched for 3 bacterial taxa compared to a high-density microcosm. Fescue with a low density (one plant) was enriched for 8 bacterial taxa compared to medium (24 plants) and high density (48 plants) microcosms. Fescue with a medium density was enriched for 4 bacterial taxa compared to low density microcosms. Fescue with a high density was enriched for 5 bacterial taxa compared to low density microcosms.

**Table 1:** Differential abundance of monoculture.

Alfalfa	Enrich group (bolded)	Brassica	Enrich group (bolded)	Fescue	Enrich group (bolded)
*Tumebacillus flagellates*	**A1** vs A24	*Bacillus mannanilyticus*	**B1** vs B48	*Devosia riboflavina*	**F1** vs F24
*Altererythrobacter dongtanensis*	**A1** vs A24	*Oscillatoria nigro-viridis*	**B1** vs B48	*Janthinobacterium* sp. LM6	**F1** vs F24
*Paenarthrobacter nicotinovorans*	**A1** vs A24	*Anabaena cylindrica*	**B1** vs B48	*Microvirga soli*	**F1** vs F24
*Devosia riboflavina*	**A1** vs A24	*Daejeonella composti*	**B1** vs B48	*Paenarthrobacter nicotinovorans*	**F1** vs F48
*Yonghaparkia alkaliphile*	**A1** vs A24	*Trichocoleus desertorum*	**B1** vs B48	*Bacillus subtilis*	**F1** vs F48
*Janthinobacterium* sp. LM6	**A1** vs A24	*Azospirillum* sp. TSA2s	**B1** vs B48	*Thermomonas* sp. SY21	**F1** vs F48
*Paenarthrobacter histidinolovorans*	**A1** vs A24	*Cyanothece* sp. PCC 7425	**B1** vs B48	*Pontibacter amylolyticus*	**F1** vs F48
*Noviherbaspirillum agri*	**A1** vs A24	*Azospirillum* sp. TSH58	**B24**-B1	*Azohydromonas australica*	**F1** vs F48
*Fictibacillus phosphorivorans*	**A1** vs A48	*Arthrobacter* sp. FB24	**B24**-B1	*Stenotrophomonas* sp. DAIF1	**F24** vs F1
*Bacillus acidicola*	**A1** vs A48	*Arthrobacter crystallopoietes*	**B24**-B1	*Roseomonas aestuarii*	**F24** vs F1
*Geitlerinema* sp. PCC 7407	**A1** vs A48	*Oscillatoria nigro-viridis*	**B24**-B48	*Ammoniphilus oxalaticu*s	**F24** vs F1
*Ammoniphilus oxalaticus*	**A1** vs A48	*Azospirillum* sp. TSA2s	**B24**-B48	*Chryseolinea soli*	**F24** vs F1
*Bacillus carboniphilus*	**A1** vs A48	*Azospirillum lipoferum*	**B24**-B48	*Stenotrophomonas* sp. DAIF1	**F48** vs F1
*Oscillatoria nigro-viridis*	**A1** vs A48			*Ammoniphilus oxalaticu*s	**F48** vs F1
*Altererythrobacter dongtanensis*	**A1** vs A48			*Telluribacter humicola*	**F48** vs F1
*Paenisporosarcina indica*	**A1** vs A48			*Roseomonas aestuarii*	**F48** vs F1
*Ammoniphilus resinae*	**A1** vs A48			*Ensifer adhaerens*	**F48** vs F1
*Bacillus subtilis*	**A1** vs A48				
*Oxalophagus oxalicus*	**A1** vs A48				
*Roseimicrobium gellanilyticum*	**A1** vs A48				
*Lysobacter helvus*	**A1** vs A48				
*Anabaena cylindrica*	**A1** vs A48				
*Bacillus carboniphilus*	**A24**-A48				
*Geitlerinema* sp. PCC 7407	**A24**-A48				
*Glaciimonas singularis*	**A24**-A48				
*Paenisporosarcina indica*	**A24**-A48				
*Larkinella harenae*	**A24**-A48				
*Oscillatoria nigro-viridis*	**A24**-A48				
*Roseimicrobium gellanilyticus*	**A24**-A48				
*Brevifollis gellanilyticus*	**A24**-A48				
*Bacillus subtilis*	**A24**-A48				
*Telluribacter humicola*	**A48**-A1				
*Ensifer adhaerens*	**A48**-A1				
*Daejeonella oryzae*	**A48**-A1				
*Azospirillum* sp. TSA2s	**A48**-A1				
*Tumebacillus flagellates*	**A48**-A24				
*Devosia riboflavina*	**A48**-A24				
*Ensifer adhaerens*	**A48**-A24				
*Paenarthrobacter nicotinovorans*	**A48**-A24				

### Mixtures of two plants

#### Plant biomass for two plant mixtures

**Figure 2:**
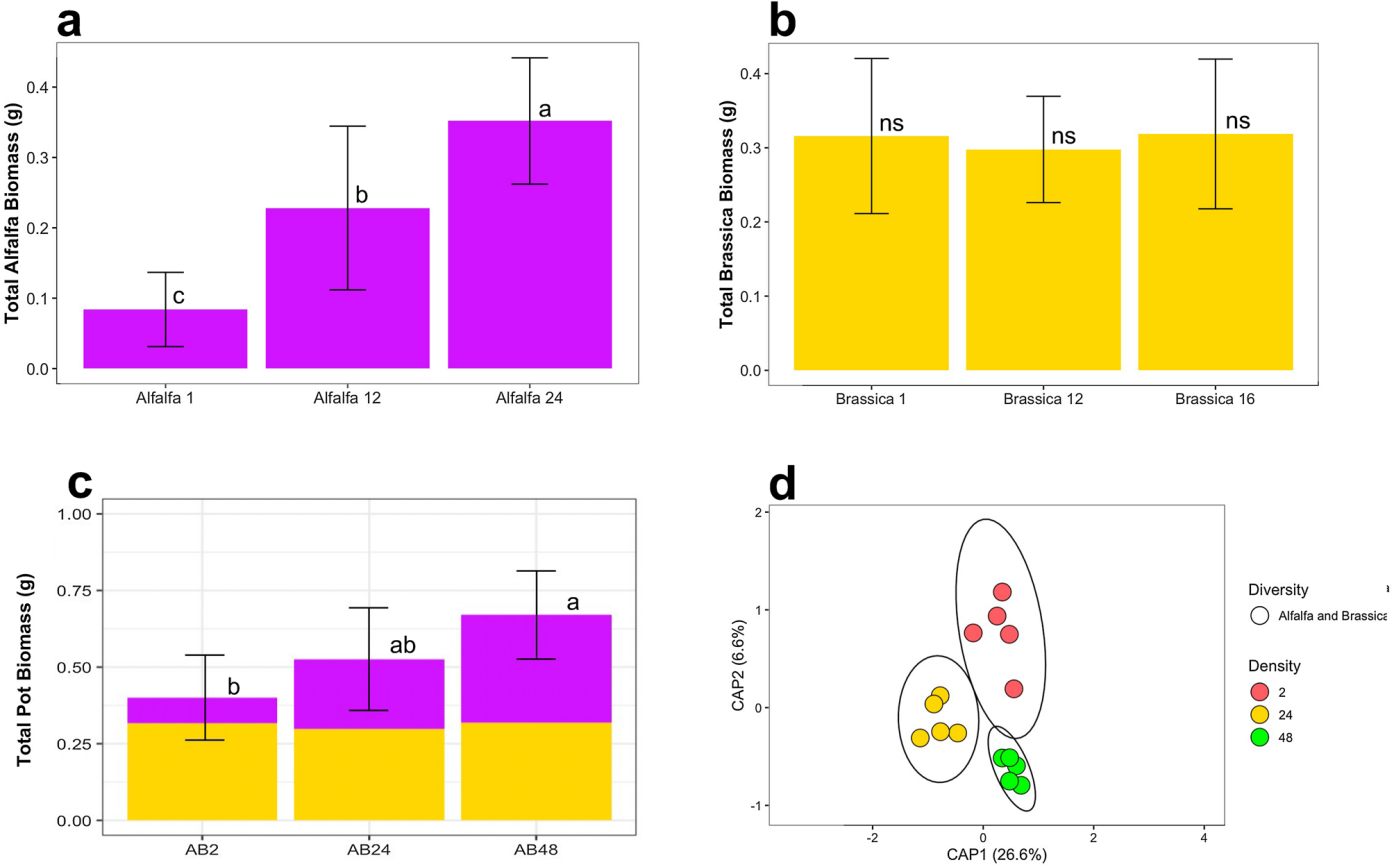
Above ground dry biomass for the alfalfa and brassica crop mixture densities of two plants total, 24 plants total, and 48 plants by (**a)** total alfalfa (purple), (**b**) total brassica (gold), and (**c**) total biomass (alfalfa and brassica). (**d**) Constrained Principal Coordinate Analysis (PCoA) using Bray–Curtis distance for comparing bulk soil bacteriomes of increasing crop densities of the alfalfa and brassica mixture. Colors were used to represent increasing densities from 2 plants (red), 24 plants (yellow), and 48 plants (green). Letters (a, b, and c) indicate significant differences between the mean values of plant biomass with (Tukey *P* < 0.05), and ns = not significant differences. Error bars are the SD.

**Figure 3:**
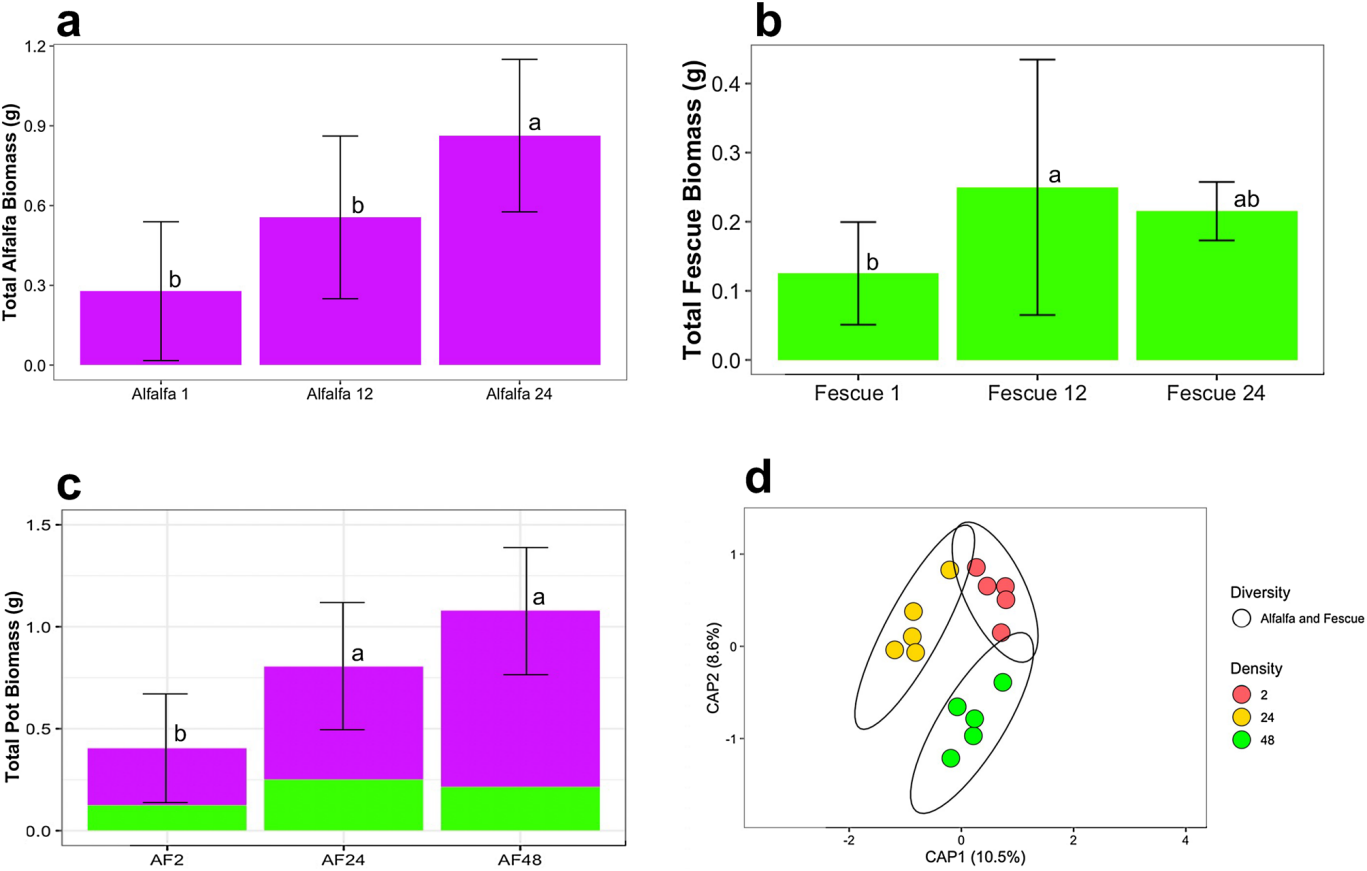
Above ground dry biomass for the alfalfa and fescue crop mixture densities of two plants total, 24 plants total, and 48 plants by (**a**) total alfalfa (purple), (**b**) total fescue (green), and (**c**) total biomass (alfalfa and fescue). (**d**) Constrained Principal Coordinate Analysis (PCoA) using Bray–Curtis distance for comparing bulk soil bacteriomes of increasing crop densities of the alfalfa and fescue mixture. Colors were used to represent increasing densities from 2 plants (red), 24 plants (yellow), and 48 plants (green). Letters (a, b, and c) indicate significant differences between the mean values of plant biomass with (Tukey *P* < 0.05). Error bars are the SD.

**Figure 4:**
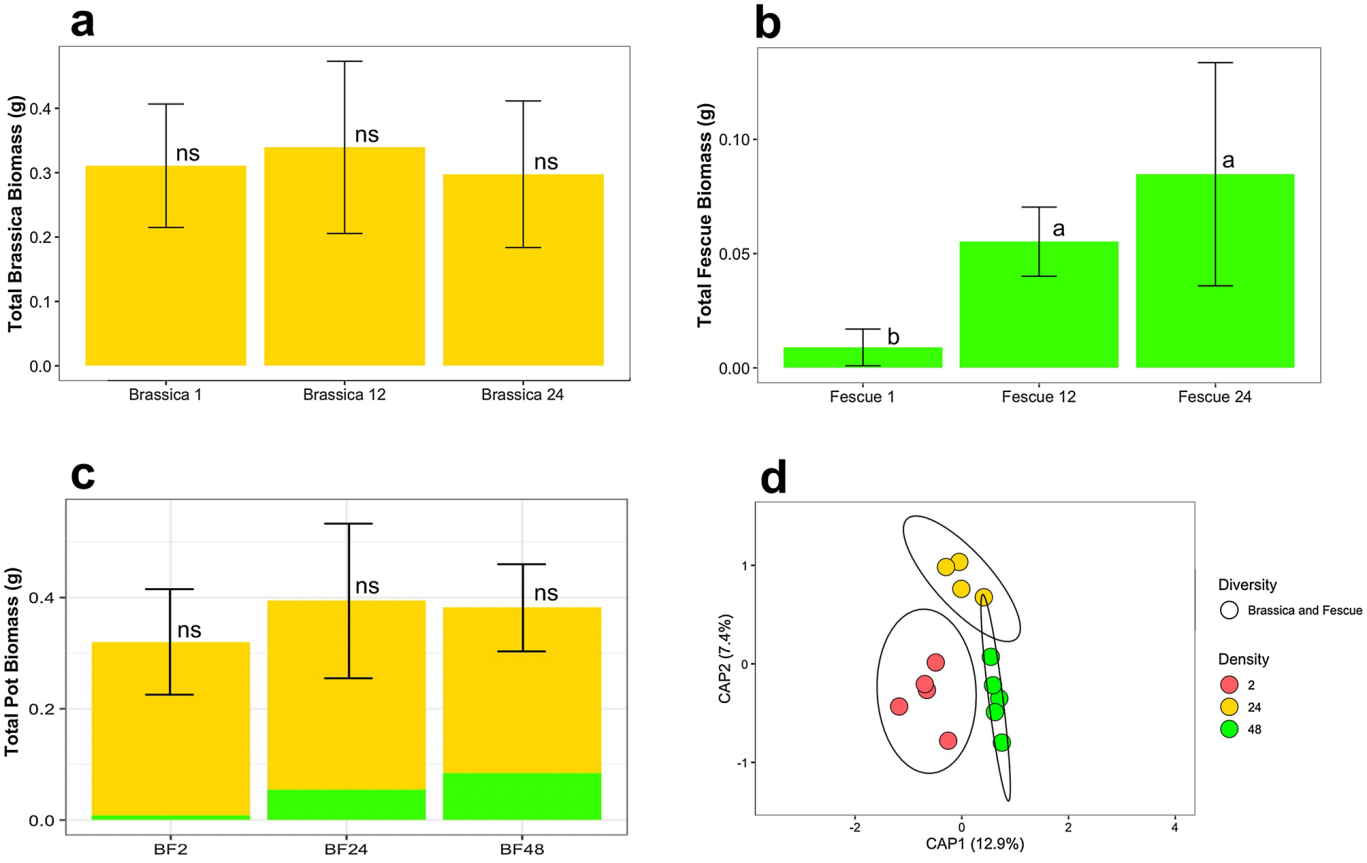
Above ground dry biomass for the alfalfa and fescue crop mixtures for densities of two plants total, 24 plants total, and 48 plants by (**a**) total brassica (gold), (**b**) total fescue (green), and (**c**) total brassica and fescue. (**d**) Constrained Principal Coordinate Analysis (PCoA) using Bray–Curtis distance for comparing bulk soil bacteriomes of increasing crop densities of the brassica and fescue mixture. Colors were used to represent increasing densities from 2 plants (red), 24 plants (yellow), and 48 plants (green). Letters (a, b, and c) indicate significant differences between the mean values of plant biomass with (Tukey *P* < 0.05), and ns = not significant differences. Error bars are the SD.

Alfalfa plant biomass increased when grown in polyculture with higher densities of brassica (Fig. 2a) or fescue (Fig. 3a). Fescue’s biomass did not significantly increase in densities of 24 and 48 plants with either alfalfa or brassica in paired mixtures (Figs. 3b and 4b). However, fescue had the highest biomass in a cover crop mixture with just alfalfa (Fig. 3b). The trend of fescue biomass in a cover crop mixture with alfalfa was similar to fescue growing in monoculture (Figs. 1e, 3b, 4b), where there was a significant increase followed by a leveling off in crop biomass. Brassica’s biomass in cover crop mixtures did not change with increasing densities (Figs. 2b, 4a). Overall, the average total above ground dry biomass was highest in the alfalfa and fescue cover crop mixture at a density of 48 plants.

#### Surrounding bulk soil bacteriome analysis for two plant mixtures

For Shannon Diversity Index, increasing plant-plant intra/inter specific competition did not increase microbial alpha diversity in the surrounding bulk soil (Supplementary Fig. S4). The PERMANOVA test showed that alfalfa and brassica mixtures under increasing densities of 2, 24, and 48 plants, induced a significant shift on the soil bacteriome (*p* = 0.01, R^2^ = 0.332) (Fig. 2d, Supplementary Table S2). For alfalfa and brassica mixtures, the average distance to the median for the bacterial microbiomes at a densities of 2 (0.305), 24 (0.272), and 48 (0.2586) plant mixtures had the highest clustering for the 48-plant density (Fig. 2d). Bacteriomes of alfalfa and brassica mixtures showed decreasing dispersion as density increased. The PERMANOVA test showed that when looking at alfalfa and fescue mixtures, increasing densities of 2, 24, and 48 plants induced a significant shift on the soil bacteriome (*p* = 0.02, R^2^ = 0.192, Supplementary Table S2) (Fig. 3d). For alfalfa and fescue mixtures, the average distance to median for the bacterial microbiomes at a density of two plant mixtures (0.3073), 24 plants (0.3067), and 48 plants (0.3240) had the highest clustering for the 24-plant density. The PERMANOVA test showed that when looking at brassica and fescue mixture by increasing densities of 2, 24, and 48 plants, the shift induced on the soil bacteriome was significant (*p* = 0.038, R^2^ = 0.203) (Fig. 4d, Supplementary Table S2). For brassica and fescue mixtures, the average distance to median for the bacterial microbiomes at a density of two plant mixtures (0.2849), 24 plants (0.2571), and 48 plants (0.2755) had the highest clustering for the 24-plant density. The increasing density of the alfalfa and brassica crop mixtures explained the highest variability (CAP1 + CAP2: 33.2) as compared to alfalfa and fescue (19.1), and brassica and fescue (20.3) (Figs. 2d, 3d, 4d).

Differential abundance analysis of the bacterial microbiome in the bulk soil was performed only if there was a change in the total biomass of a crop within the mixture (Table 2). Alfalfa and brassica mixture in low density (2 plants) showed an enrichment of 26 bacterial taxa compared and high density (48 plants) microcosms whereas high compared to low densities showed an enrichment of 2 bacterial taxa. Alfalfa and fescue mixture in low density (2 plants) showed an enrichment of 8 bacterial taxa compared to high (48 plants) and medium density (24 plants) microcosms whereas high and medium densities showed an enrichment of 13 bacterial taxa as compared to low density microcosms. There was no biomass increase for the total biomass for brassica and fescue mixture, and the biomass change for fescue was used instead to highlight bacteria with significant differential abundances. Brassica and fescue mixture in low density (two plants) showed an enrichment of 8 bacterial taxa compared to high density (48 plants) microcosms whereas high densities showed an enrichment of 1 bacterial taxon as compared to low density microcosms. Brassica and fescue mixture in medium density (24 plants) showed an enrichment of 5 bacterial taxa compared to high density (48 plants) microcosms whereas high densities showed an enrichment of 6 bacterial taxa as compared to medium density microcosms.

**Table 2:** Differential abundance of two plant mixtures.

Alfalfa–Brassica	Enrich group (bolded)	Alfalfa–Fescue	Enrich group (bolded)	Brassica–Fescue	Enrich group (bolded)
*Achromobacter spanius*	**AB2** vs AB48	*Paenarthrobacter histidinolovorans*	**AF2** vs AF24	*Paenibacillus agaridevorans*	**BF2** vs BF48
*Achromobacter xylosoxidans*	**AB2** vs AB48	*Telluribacter humicola*	**AF2** vs AF24	*Oscillatoria nigro-viridis*	**BF2** vs BF48
*Achromobacter insolitus*	**AB2** vs AB48	*Lysobacter helvus*	**AF2** vs AF24	*Vicinamibacter silvestris*	**BF2** vs BF48
*Stenotrophomonas* sp. G4	**AB2** vs AB48	*Chryseolinea soli*	**AF2** vs AF48	*Pontibacter brevis*	**BF2** vs BF48
*Agrobacterium tumefaciens*	**AB2** vs AB48	*Azospirillum* sp. TSA2s	**AF2** vs AF48	*Thermomona*s sp. SY21	**BF2** vs BF48
*Geitlerinema* sp. PCC 7407	**AB2** vs AB48	*Brevibacillus brevis*	**AF2** vs AF48	*Brevifollis gellanilyticus*	**BF2** vs BF48
*Daejeonella oryzae*	**AB2** vs AB48	*Sandaracinus amylolyticus*	**AF2** vs AF48	*Anabaena cylindrica*	**BF2** vs BF48
*Paenarthrobacter nicotinovorans*	**AB2** vs AB48	*Thermomona*s sp. SY21	**AF2** vs AF48	*Trichocoleus desertorum*	**BF2** vs BF48
*Kaistia defluvii*	**AB2** vs AB48	*Achromobacter insloitus*	**AF24** vs AF2	*Pontibacter brevis*	**BF24** vs BF48
*Telluribacter humicola*	**AB2** vs AB48	*Stenotrophomonas* sp. MYb57	**AF24** vs AF2	*Paenibacillus agaridevorans*	**BF24** vs BF48
*Adhaeribacter swui*	**AB2** vs AB48	*Achromobacter xylosoxidans*	**AF24** vs AF2	*Vicinamibacter silvestris*	**BF24** vs BF48
*Paucimonas lemoignei*	**AB2** vs AB48	*Stentotrophomona*s sp. DAIF1	**AF24** vs AF2	*Thermomonas* sp. SY21	**BF24** vs BF48
*Roseomonas aestuarii*	**AB2** vs AB48	*Agrobacterium tumefaciens*	**AF24** vs AF2	*Trichocoleus desertorum*	**BF24** vs BF48
*Chryseolinea soli*	**AB2** vs AB48	*Azospirillum* sp. TSH58	**AF24** vs AF2	*Pontibacter chitinilyticus*	**BF48** vs BF2
*Altererythrobacter dongtanensis*	**AB2** vs AB48	*Anabaena cylindrica*	**AF24** vs AF2	*Azospirillum brasilense*	**BF48** vs BF24
*Vicinamibacter silvestris*	**AB2** vs AB48	*Luteolibacter pohnpeiensis*	**AF24** vs AF2	*Paenarthrobacter nicotinovorans*	**BF48** vs BF24
*Trichocoleus desertorum*	**AB2** vs AB48	*Achromobacter insloitus*	**AF48** vs AF2	*Ensifer adharenes*	**BF48** vs BF24
*Janthinobacterium* sp. LM6	**AB2** vs AB48	*Achromobacter xylosoxidans*	**AF48** vs AF2	*Pontibacter chitinlyticus*	**BF48** vs BF24
*Anabaena cylindrica*	**AB2** vs AB48	*Agrobacterium tumefaciens*	**AF48** vs AF2	*Larkinella harenae*	**BF48** vs BF24

### Mixtures of three plants

#### Plant biomass of mixture

**Figure 5:**
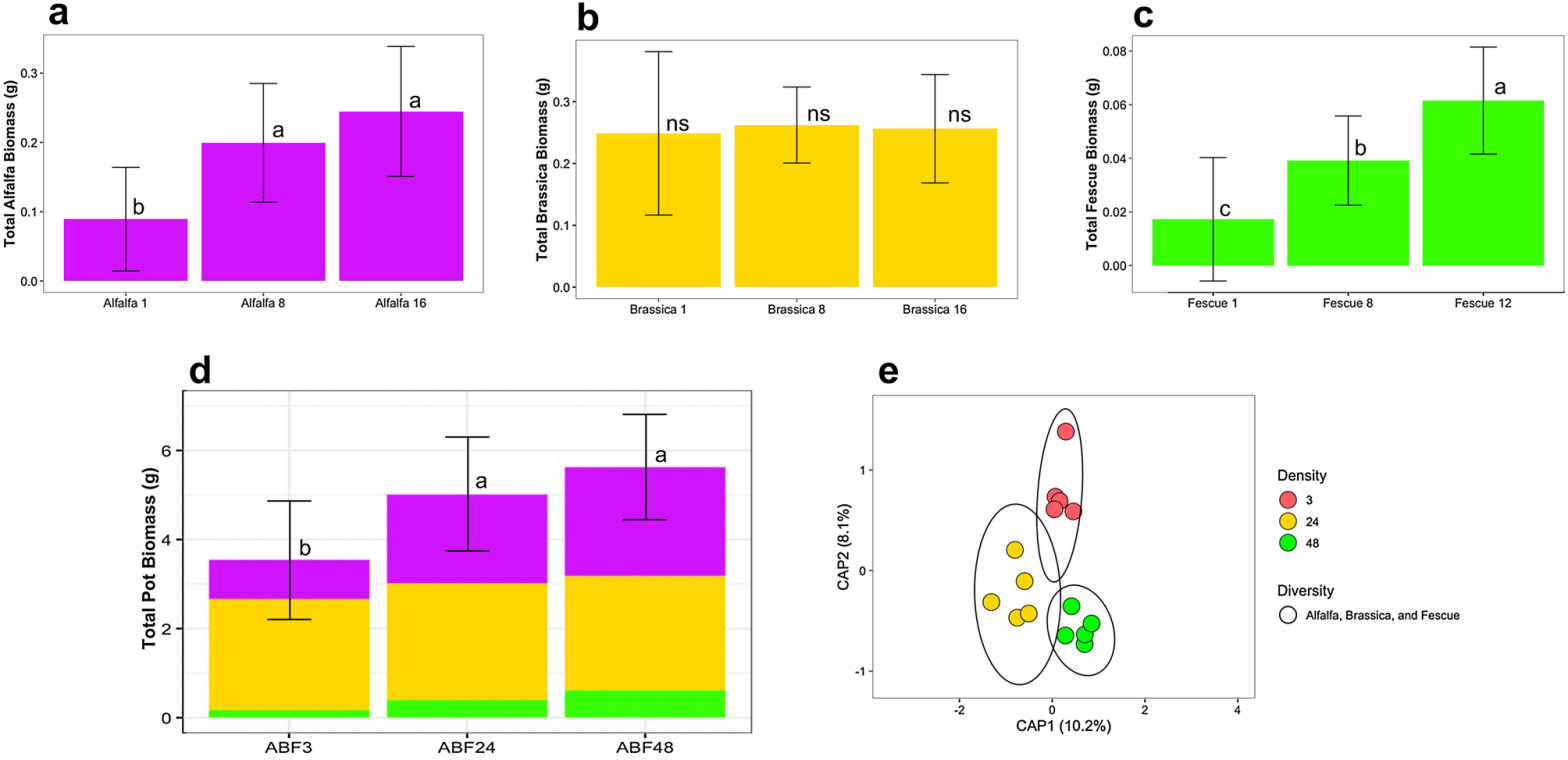
Above ground dry biomass for the alfalfa, brassica, and fescue crop mixture densities of three plants total, 24 plants total, and 48 plants shown separately by (**a**) total alfalfa (purple), total brassica (gold), total fescue (green), and (**b**) total biomass (alfalfa, brassica, and fescue). (**d**) Constrained Principal Coordinate Analysis (PCoA) using Bray–Curtis distance for comparing bulk soil bacteriomes of increasing crop densities of the alfalfa, brassica, and fescue mixture. Colors were used to represent increasing densities from 3 plants (red), 24 plants (yellow), and 48 plants (green). Letters (a, b, and c) indicate significant differences between the mean values of plant biomass with (Tukey *P* < 0.05), and ns = not significant differences. Error bars are the SD.

The mixture with three different cover crops showed similar trends as when they were grown in the cover crop mixtures of just two crops. When all three plants were grown together, there was a higher biomass for alfalfa and fescue as density increased, while there was no increase in biomass for brassica (Fig. 5b). For alfalfa, this trend was different that the previous cover crop mixtures and in monoculture since the density increase of 24 to 48 plants did not show an increase in biomass. Fescue biomass remained as the lowest in the mixture of three crops (Fig. 5c). The number of brassica plants did not influence the total amount of above ground biomass for brassica. In summary, the biomass trends of mixtures of three cover crops followed previous trends for the mixtures of two cover crops.

#### Surrounding bulk soil bacteriome analysis

Biomass of an individual plant was largest in the density of three plants and decreased as density increased (Fig. 3c) Mixtures with all crops of a low density (3 plants) to medium density (24 plants) both showed an enrichment of 1 taxon (Table 3). Mixtures with all crops of a low density (3 plants) to high density (48 plants) showed an enrichment of 11 taxa, while high densities had an increase of 2 taxa as compared to low densities (Table 3). The PERMANOVA test showed that when looking at fescue by increasing densities of 3, 24, and 48 plants, the shift induced on the soil bacteriome was not significant (*p* = 0.069, R^2^ = 0.183) (Fig. 5e, Supplementary Table S2). For the mixture of all three plants, the average distance to median for the bacterial microbiomes at a density of three plants (0.3123), 24 plants (0.3035), and 48 plants (0.3279) had the highest clustering for 24 plants.

**Table 3:** Differential abundance of 3 plant mixtures.

Alfalfa–Brassica–Fescue	Enrich group (bolded)
*Paenisporosarcina indica*	**ABF3** vs ABF24
*Trichocoleus desertorum*	**ABF3** vs ABF48
*Stenotrophomonas* sp. DAIF1	**ABF3** vs ABF48
*Geitlerinema* sp. PCC 7407	**ABF3** vs ABF48
*Azospirillum brasilense*	**ABF3** vs ABF48
*Azospirillum* sp. TSH58	**ABF3** vs ABF48
*Devosia riboflavina*	**ABF3** vs ABF48
*Oscillatoria nigro-viridis*	**ABF3** vs ABF48
*Noviherbaspirillum denitrificans*	**ABF3** vs ABF48
*Arcticibacter svalbardensis*	**ABF3** vs ABF48
*Brevifollis gellanilyticus*	**ABF3** vs ABF48
*Azospirillum* sp. TSA2s	**ABF3** vs ABF48
*Planctomyces* sp. SH-PL14	**ABF24** vs ABF3
*Planctomyces* sp. SH-PL14	**ABF48** vs ABF3
*Sandaracinus amylolyticus*	**ABF48** vs ABF3

## Discussion

In this study, it was shown that *Azospirillum* sp. TSA2s and *Ensifer adhaerens* increased between single to and 48 plants, while *Devosia riboflavina* and *E. adhaerens* increased between alfalfa densities of 24 and 48 plants. *Azospirilum sp*. and *E. adhaerens* are free-living nitrogen fixers, and *D. riboflavina* is a weak nitrate reducer; these species play a role in the nitrogen cycle in legume inhabited soils^33–37^. Alfalfa’s monoculture exhibited the least dispersion at densities of 48 plants, suggesting that the surrounding bulk soil bacteriome is progressing towards a tailored microbiome for alfalfa as intraspecific competition increases.

Though brassicas are known to produce antimicrobials^15^, the Shannon Index did not reflect a decrease in of bacterial taxa as density increased. Taye et al., 2020^38^ found that *Brassica napus* recruited many bacterial taxa whose effects included disease suppression. Incidentally, the abundance of the antiprotozoal microbe *Oscillatoria nigro-viridis* increased between brassica densities of 24 and 48 plants^39^. It was previously found that intraspecific competition in *B. juncea* manifested as increased counts of stress induced inflorescences and bolting^40,41^. In the present study, brassica bolted at the 24 and 48 plant densities which could be attributed to increasing intraspecific competition. Nitrogen might be in demand for competing brassica plants, since increasing densities were correlated with nitrogen fixers like *Azospirillum* sp. (TSH58) between single and 48 brassica densities, along with *Azospirillum* sp. TSA2s and *Azospirillum lipoferum* for brassica densities of 24–48^33,42^. *Azospirillum* sp. are also known to produce phytohormones^43^. These shifts in the bacterial composition could potentially lower plant intraspecific competition by increasing the availability of limited nutrients.

Previous studies found that under increased *Festuca* spp. densities, seed germination remained high while plant mortality decreased. The same study found that increased *Festuca* spp. density did not increase the total biomass, which could be explained by intraspecific competition^44^. It has also been reported that *Festuca* sp. density was directly correlated with the infection rate of the fungal pathogen *Rhizoctonia solani*^45^. In contrast, beneficial bacteria identified in the present study, such as *Roseomonas aestuarii* (can produce indole from tryptophan), increased from single to 24 and 48 fescue densities^46^. From a density of a single fescue plant to 48 plants the nitrogen fixer *E. adhaerens* increased similar to alfalfa. *Stenotrophomonas* sp. (DAIF1), a possible bacterial phytopathogen, also increased from single to 24 and 48 fescue densities^47,48^. However, *Stenotrophomonas* sp. has also been found to be beneficial by providing stress protection, growth promotion, and biocontrol for plants^49^. While beneficial bacteria like *E. adhaerens* could reduce plant–plant intraspecific competition, the present study has shown that higher fescue densities fail to increase total fescue biomass, indicating that there are other factors such as an asymptomatic phytopathogens playing a role in plant health.

In a previous study, an intercropping of alfalfa (*Medicago sativa*) and *B. juncea* showed that alfalfa’s biomass increased by 55.3–70.0% while *B. juncea* biomass decreased by 0.4–11.8% which was attributed to an increased uptake of cadmium by *B. juncea* and a decrease uptake by alfalfa as compared to when grown alone^50^. The present study does not support an increase in alfalfa biomass compared to grown in monoculture. Regardless, alfalfa’s biomass increased with plant density, while brassica’s remained the same. Although plants in the Brassicaceae family are not known to form mycorrhizal fungal connections^51^, this study supports the possibility that brassica plants rely on bacterial nitrogen fixers like *Azospirillum* spp when grown alone. It was expected that *Azospirillum* spp. would have enriched in alfalfa and brassica plant mixtures, since *Azospirillum* spp. was enriched for both alfalfa and brassica bulk soil monocultures. Instead, *Pseudarthrobacter phenanthrenivorans*, which has been known to produce numerous phytohormones (abscisic acid, auxin, cytokinin, ethylene, gibberellins, jasmonic acid, and salicylic acid), and the denitrifier *Pseudomonas stutzeri* were enriched^52,53^. This finding supports that plant-plant interaction influence microbial recruitment in its own manner.

Alfalfa (*M. sativa*) and tall fescue [*Schedonorus phoenix* (Scop.) Holub] mixtures have been found to have a higher above ground biomass accumulation and weed suppression as compared to respective monocultures in other studies^54^. In this study, alfalfa and fescue mixture at 24 and 48 densities produced the highest above ground biomass out of the three plant mixtures. *Achromobacter xylosoxidans*, which has been previously associated with grasses and is a known plant growth promoting rhizobacterium, was identified in the differential abundance analysis for the single pair densities to the 24 and 48 densities^55^. *Stentotrophomona*s spp. (genus known as PGPR and nitrogen fixers) abundance increased as previously observed in fescue monocultures, and Azopirillum sp. abundance increased as previously observed in fescue monocultures^56^. Nonetheless, increasing plant diversity has the potential to allow for generalist bacterial phytopathogens to transmit from one plant species to another^57^. This drawback could spur *Agrobacterium tumefaciens*, which was enriched in alfalfa-fescue mixtures and has been known to cause crown gall disease in numerous plants^58^. Strains of *A. tumefaciens* have shown to be highly virulent on alfalfa (*M. sativa*)^59^ and to infect *Fescue* spp.^60^. The increase of a known generalist phytopathogen was unexpected within the alfalfa-fescue mixtures since this plant mixture had the highest total biomass. Alfalfa’s biomass continued to increase and was not distinctly impacted by the potential phytopathogen, whereas a non-significant decrease was observed for the fescue biomass at the highest density.

Aqueous extracts of *B. juncea* were found to induce total inhibition of root and shoot growth in (barnyard) grass^61^. Similarly in the present study, fescue biomass was the lowest in mixtures with brassica, possibly due to interspecific competition. In brassica-fescue mixtures, *Pontibacter chitinilyticus* which has possible antifungal capabilities through chitin-hydrolsis, was enriched^62,63^. The nitrogen fixer *Azospirillum brasilense* was also relatively enriched much like when brassica was grown alone^64^. The reoccurring nitrogen fixing species *Ensifer adharenes* was correlated with the presence of fescue, and could be a bacterium which reduces plant-plant competition leading to the significant total biomass increase. Drawing from biomass trends and differential microbial abundance results, brassica did not seem to be influenced by the presence of fescue.

For the crop mixtures of all three plants, differential abundance highlighted bacterial taxa whose abundances were not different in the monocultures or two-plant mixtures. *Planctomyces* sp. SH-PL14, known for its chitinase ability, and *Sandaracinus amylolyticus*, which has exhibited both antimicrobial production and starch hydrolysis, were highlighted in differential abundance analysis when moving from the soil with three individual plants to a density of 24 or 48 total plants^65–67^. While *Planctomyces* sp. chitinase could benefit all plants by causing a decrease in fungal phytopathogens^51^, and in our study these bacteria may contribute to reducing beneficial mycorrhizal networks for both alfalfa and fescue. It is also interesting to note that this chitinase producing bacteria was not identified in monocultures of brassica, suggesting brassica may not have a need for chitinase since the plant does not promote mycorrhizal networks. It is thought that *Brassica* spp. reduce the growth of interspecific competitors by producing the allelochemical sinigrin, which reduces mycorrhizal abundance of surrounding soils. However, sinigrin production is costly, and this investment does not alleviate intraspecific competition^51^. The nitrogen fixing *Azospirillum spp.* and *E. adharenes* found in monocultures were not apparent in three-plant mixture soils. Co-existence would have been supported if free-living nitrogen fixers, even species driven dependent, was found. Abundances of the generalist phytopathogen, *Agrobacterium tumefaciens*, was not enriched in the plant mixture of the highest density and diversity, suggesting the soil bacteriome may benefit by increasing the diversity of alfalfa-fescue mixtures. This would support the dilution effect, where an increase of biodiversity decreases pathogen exposure and transmission^68,69^. Even in the most competitive mixture and density, bacterial phytopathogens were not highlighted by differential abundance. Overall, the present study shows that, while increasing densities can further increase previously promoted bacterial taxa, increasing plant diversity does not simply increase bacterial diversity as different bacterial taxa can then be promoted.

Autoclaving the soil simplifies the microbial community and has been shown to magnify the effect of plants on the soil bacteriome compared to non-autoclaved soils^25^. Bacteriome shifts in the surrounding bulk soil in the microcosms are not comparable to the greater space in the field where spatial variability and legacy effects may have an influence. Moreover, a plant’s developmental stage can influence the recruitment and selection of bacteria^70^. Additional studies are required to directly define the functionality of these bacteria which could play a moderator role in plant-plant competition. In summary, this study supports the notion that bacterial shifts in the soil could depend on plant–plant interactions. The surrounding bulk soil bacteriomes of polycultures did not completely overlap with the bacteriomes of monocultures. Thus, bacteriome functionalities are not expected to be a simple overlap when one plant species is planted with another.

### Supplementary Information

Supplementary Information.

## Data Availability

The datasets (DNA concentrations, sequencing data, plant biomass, and R code) generated and/or analyzed during the current study are available in the GitHub, [https://github.com/Derek-Newberger/ScientificReports_Newberger_BulkSoilBacteriome]. Raw data are available from the corresponding author on reasonable request.
