# Supplementary material for: Shifts of the soil microbiome composition induced by plant–plant interactions under increasing cover crop densities and diversities
Source: Sci Rep. 2023 Oct 10;13:17150. doi: 10.1038/s41598-023-44104-8 (PMC10564930; doi:10.1038/s41598-023-44104-8)
Supplement: Supplementary file 1 — Supplementary Information. [file 41598_2023_44104_MOESM1_ESM.docx]

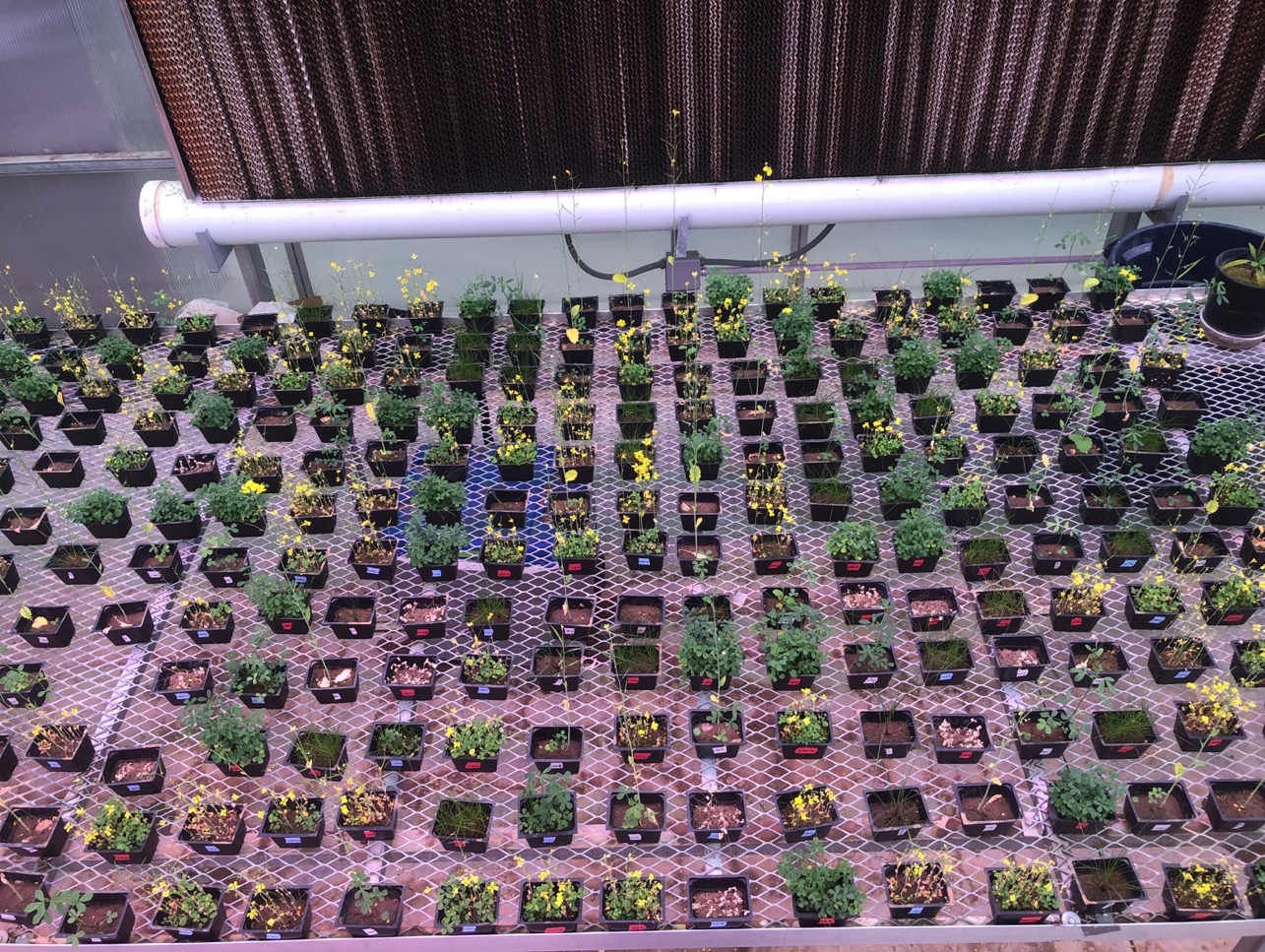


Supplementary Figure S1: Picture of the microcosm experiment

Supplementary Table S1: Experimental set up of the 21 different treatments

| \|  \| Monoculture \| Polyculture \| \| --- \| --- \| --- \| \| Low Density \| 1. Alfalfa (1 Plant) \| 10. Alfalfa (1 Plant) and Brassica (1 Plant) \| \| (1-3 Plants per microcosm) \| 2. Brassica (1 Plant) \| 11. Alfalfa (1 Plant) and Fescue (1 Plant) \| \|  \| 3. Fescue (1 Plant) \| 12. Brassica (1 Plant) and Fescue (1 Plant) \| \|  \|  \| 13. Alfalfa (1 Plant), Brassica (1 Plant), and Fescue (1 Plant) \| \| Medium Density \| 4. Alfalfa (24 Plants) \| 14. Alfalfa (12 Plants) and Brassica (12 Plants) \| \| (24 Plants per microcosm) \| 5. Brassica (24 Plants) \| 15. Alfalfa (12 Plants) and Fescue (12 Plants) \| \|  \| 6. Fescue (24 Plants) \| 16. Brassica (12 Plants) and Fescue (12 Plants) \| \|  \|  \| 17. Alfalfa (8 Plants), Brassica (8 Plants), and Fescue (8 Plants) \| \| High Density \| 7. Alfalfa (48 Plants) \| 18. Alfalfa (24 Plants) and Brassica (24 Plants) \| \| (48 Plants per microcosm) \| 8. Brassica (48 Plants) \| 19. Alfalfa (24 Plants) and Fescue (24 Plants) \| \|  \| 9. Fescue (48 Plants) \| 20. Brassica (24 Plants) and Fescue (24 Plants) \| \|  \|  \| 21. Alfalfa (16 Plants), Brassica (16 Plants), and Fescue (16 Plants) \| |
| --- | --- | --- | --- | --- | --- | --- | --- | --- | --- | --- | --- | --- | --- | --- | --- | --- | --- | --- | --- | --- | --- | --- | --- | --- | --- | --- | --- | --- | --- | --- | --- | --- | --- | --- | --- | --- | --- | --- | --- |


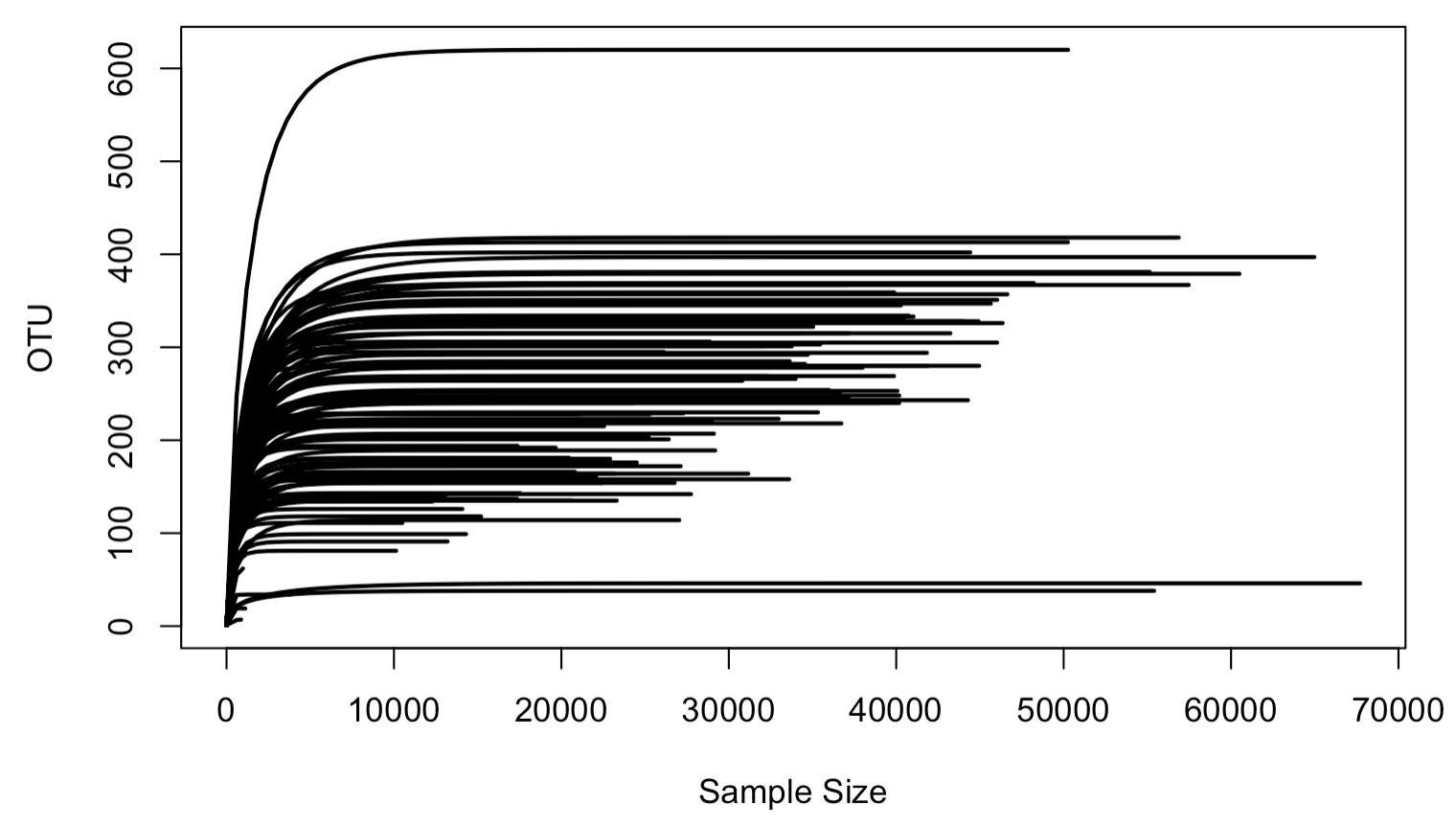


Supplementary Figure S2: Rarefaction Curve for all samples


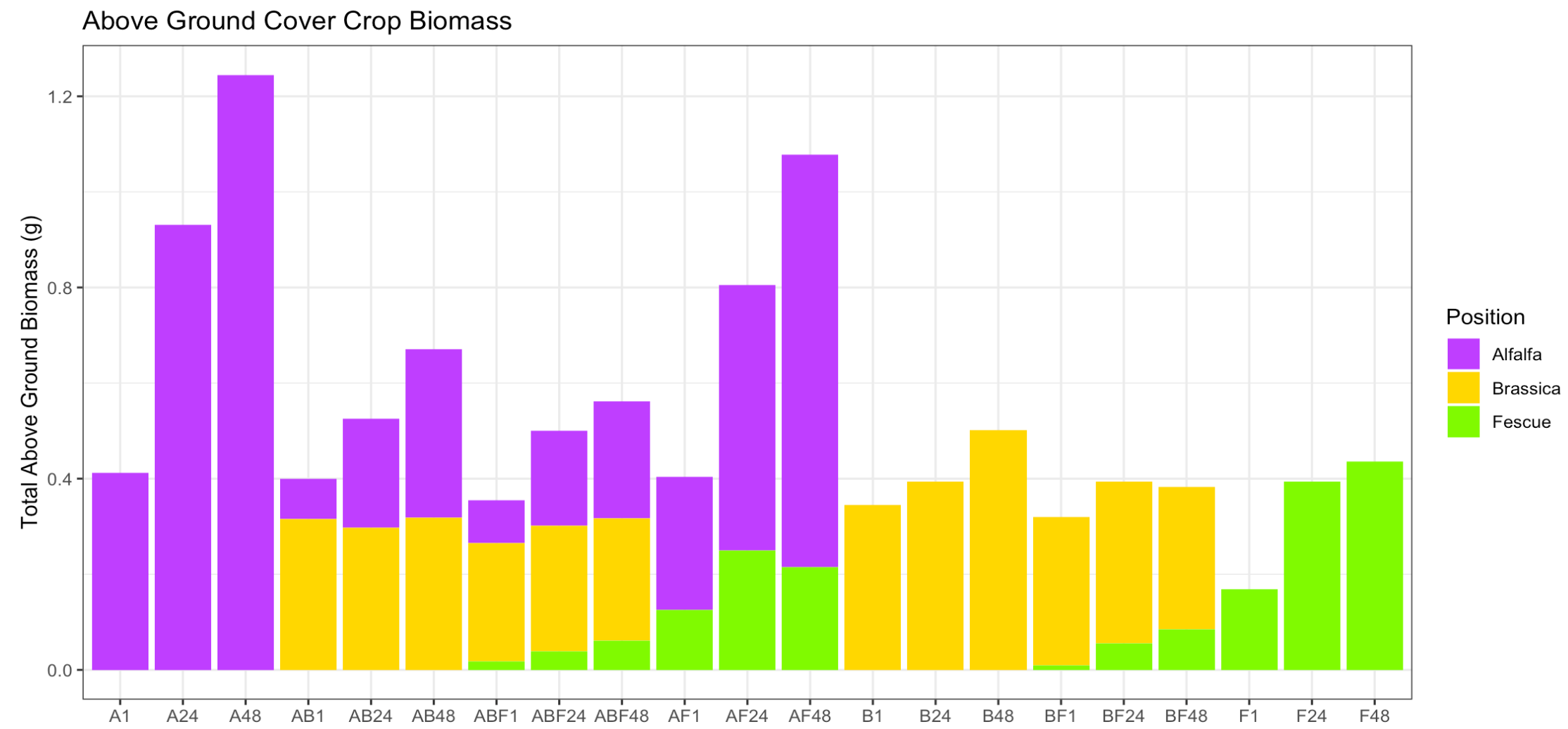


Supplementary Figure S3: Total above ground cover crop biomass stacked by crop (alfalfa: purple, brassica: gold, fescue: lime green).


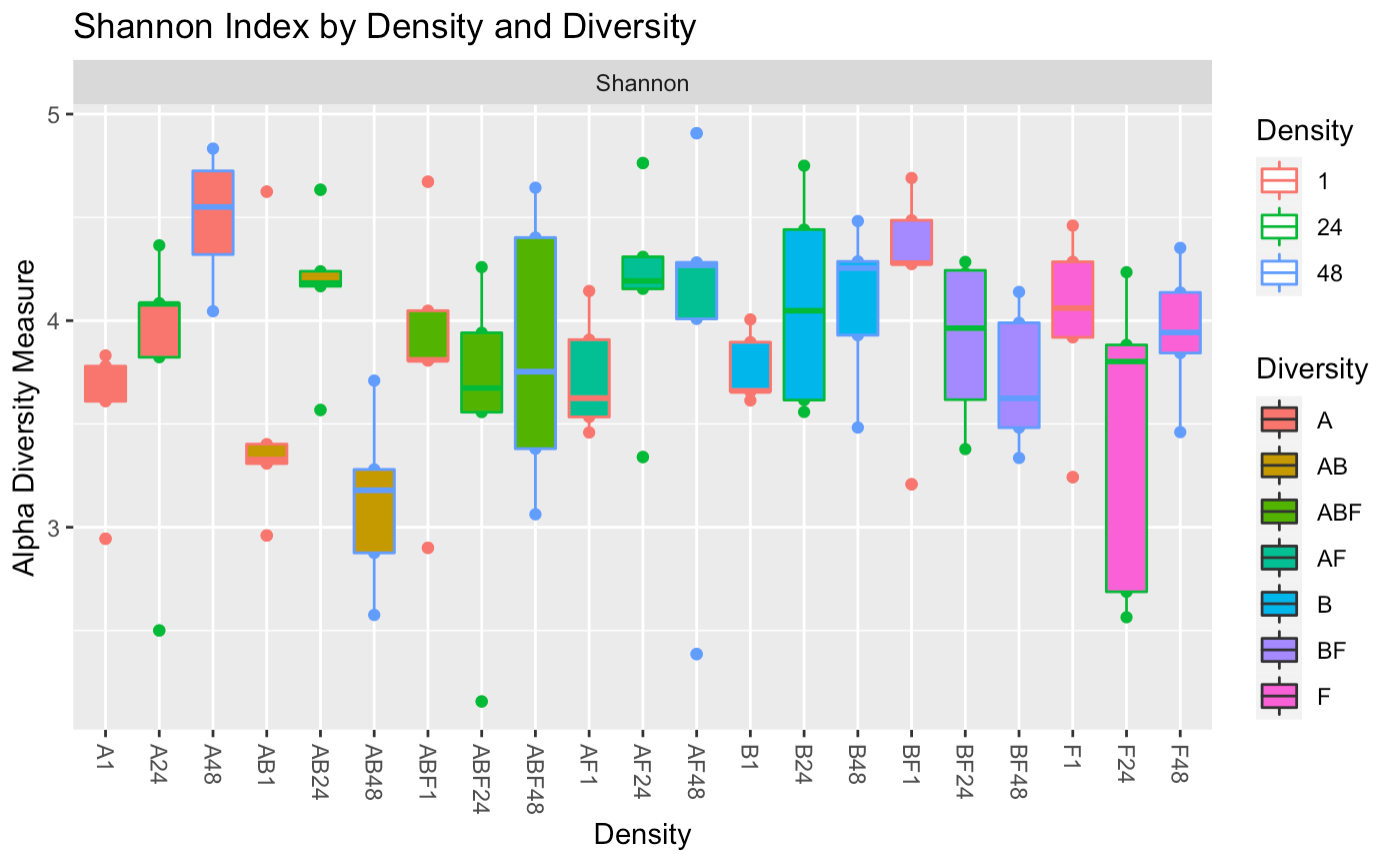


Supplementary Figure S4: Alpha diversity of bacterial microbiomes denoted by increasing densities of monoculture and plant mixtures.


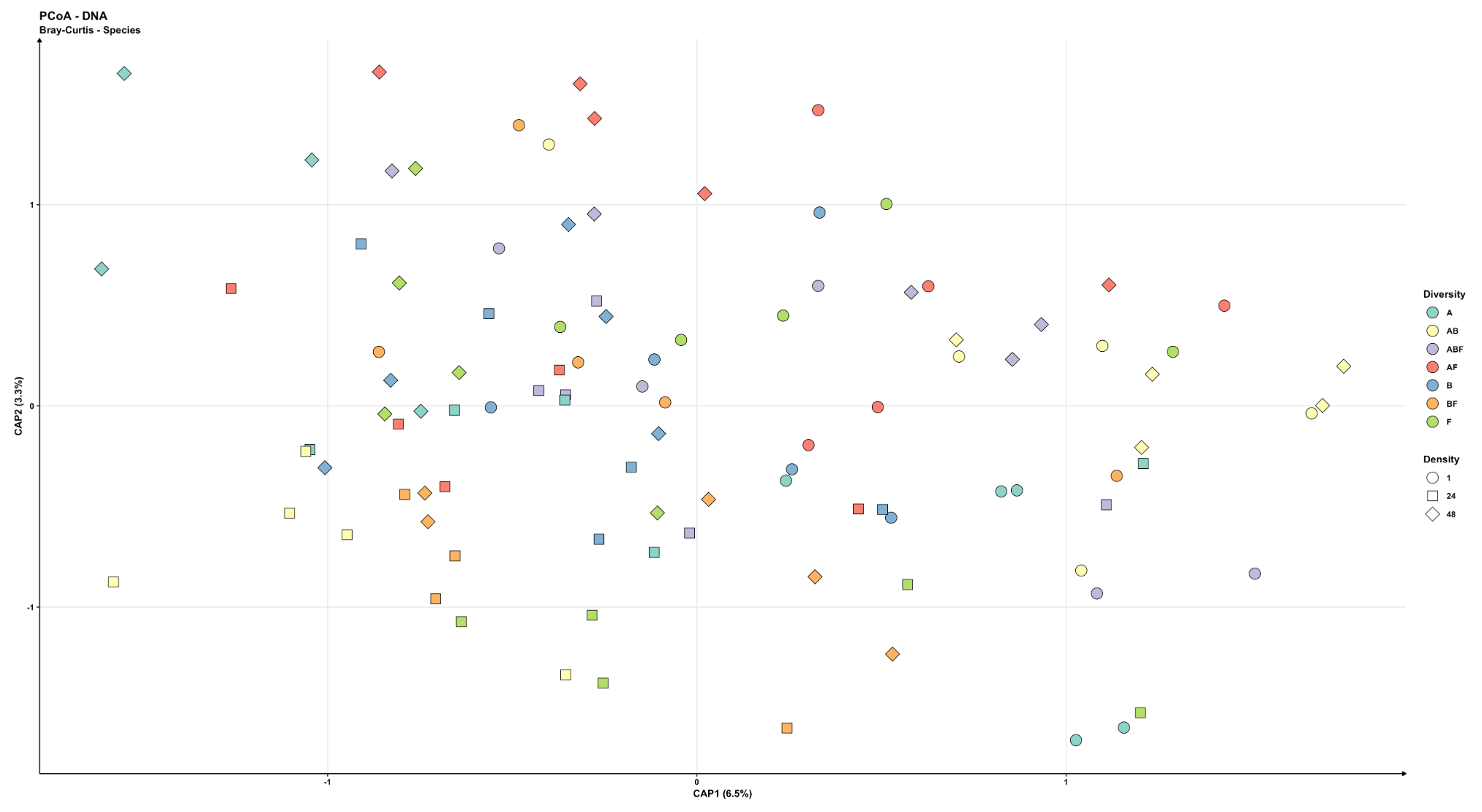


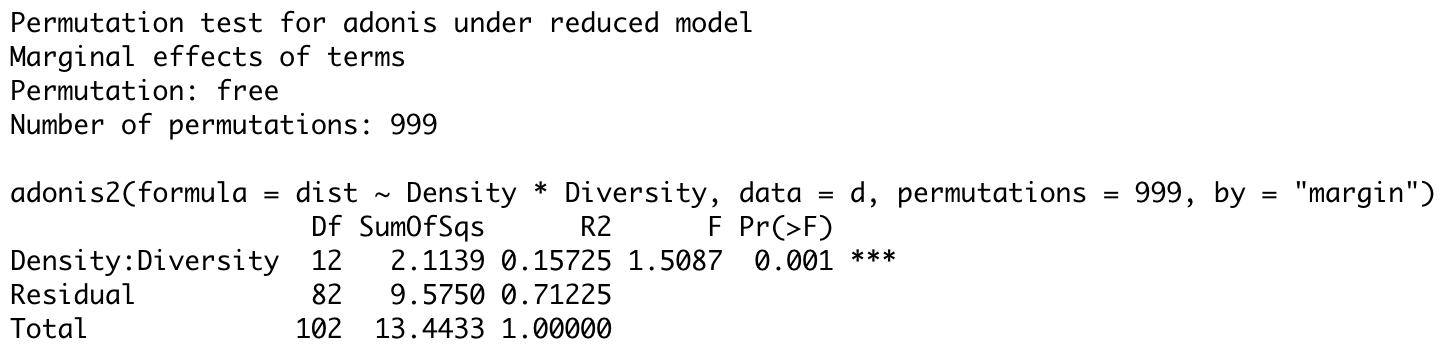

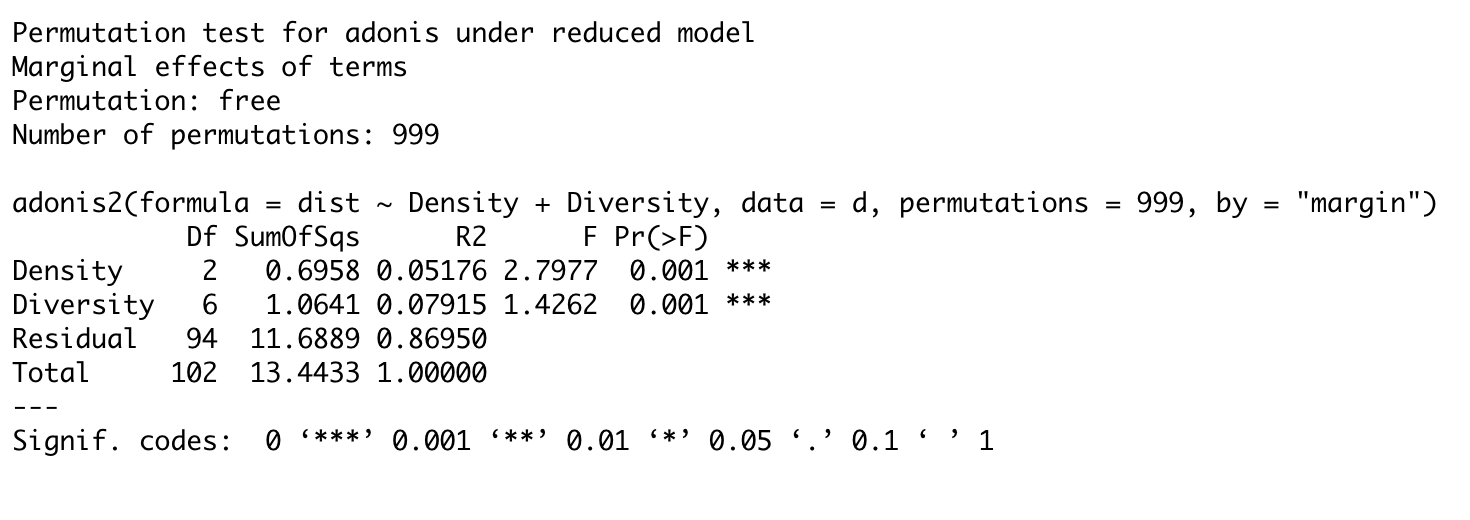


Supplementary Figure S5: PERMANOVA model with all data combined of the interaction between plant diversity and density and the significance of both factors separately on the structure of bacteriomes

Supplementary Table S2: Analysis of Principal Coordinates Summary:

Alfalfa Monocrop


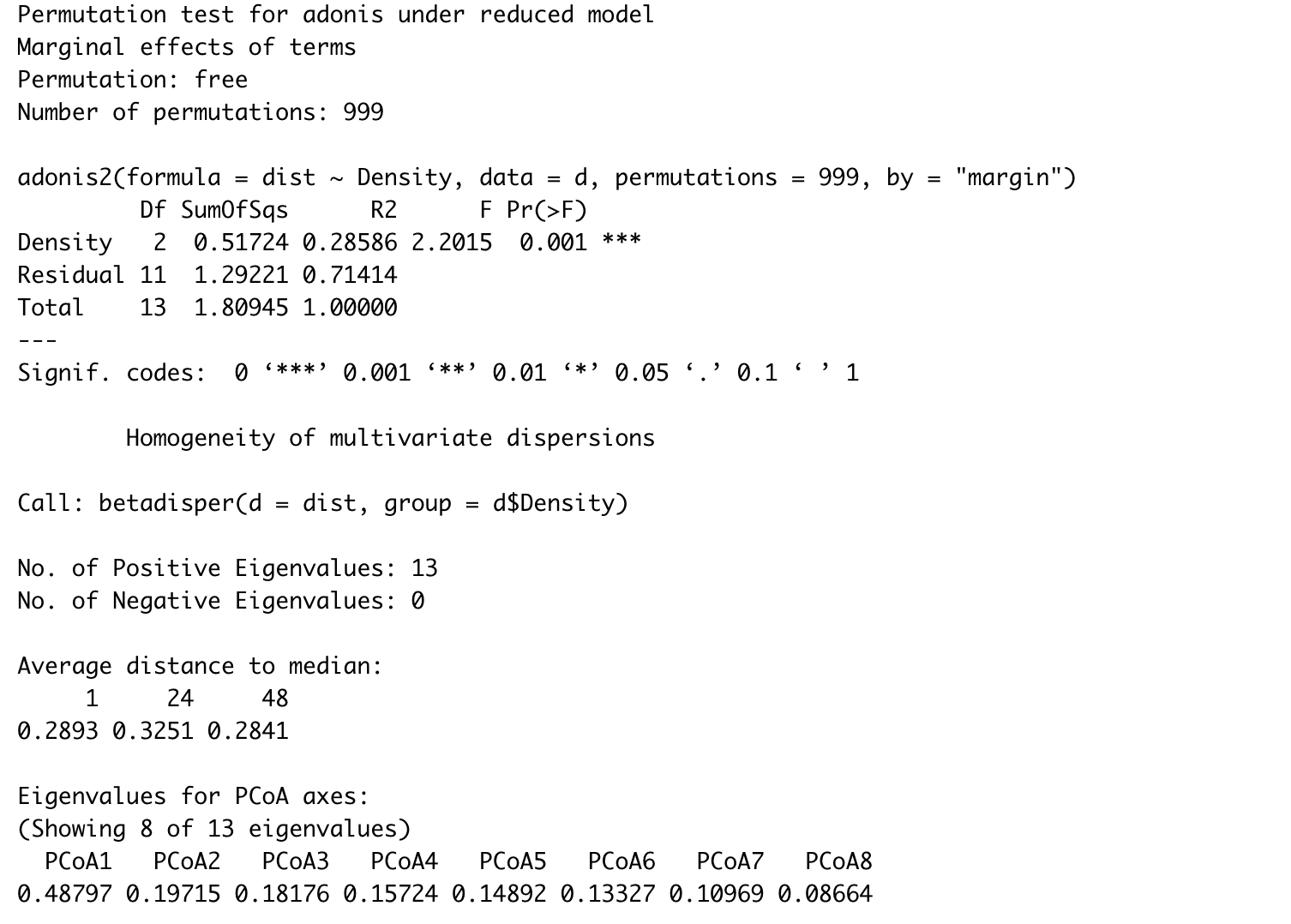


Brassica Monocrop


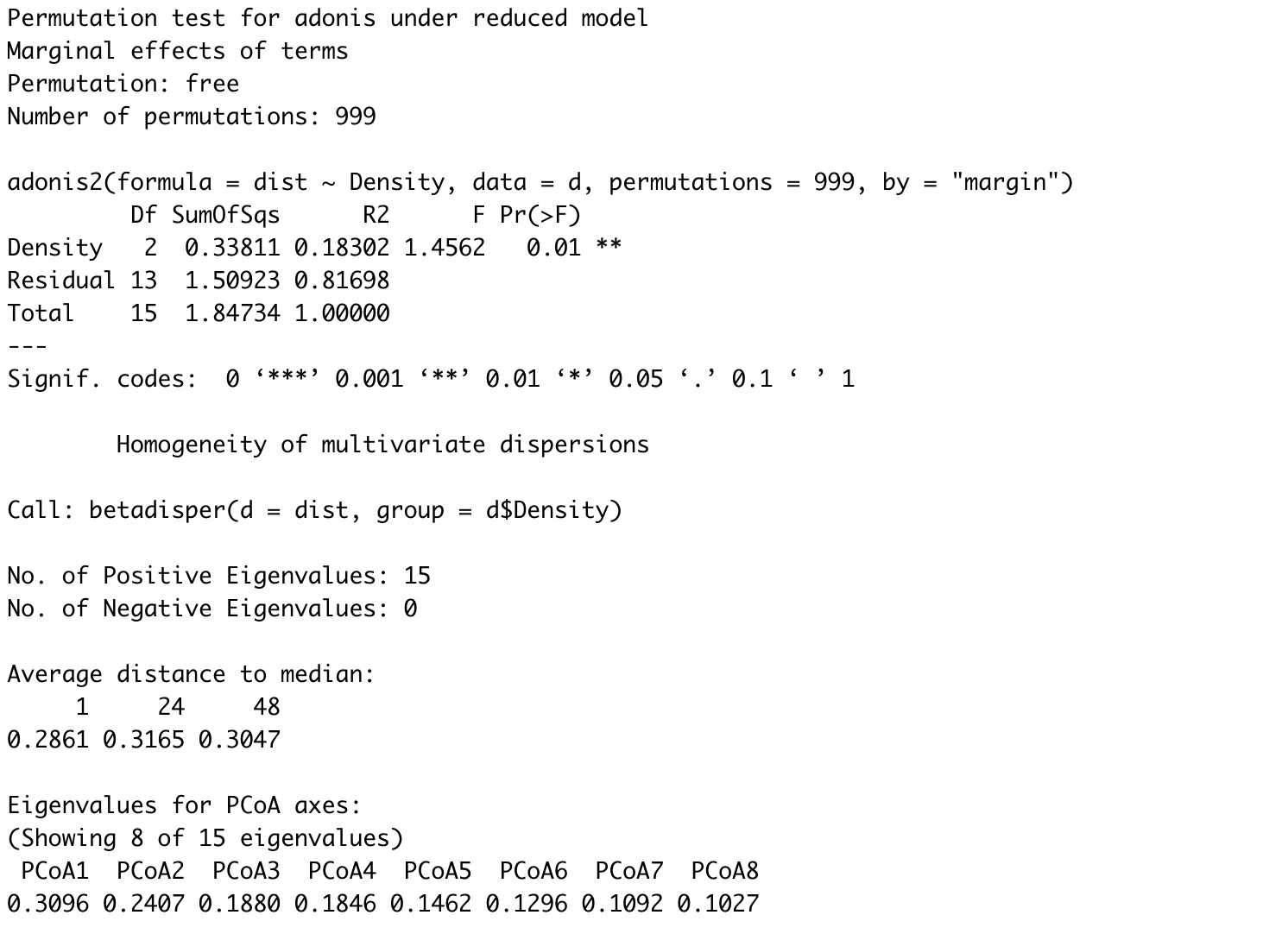


Fescue Monocrop


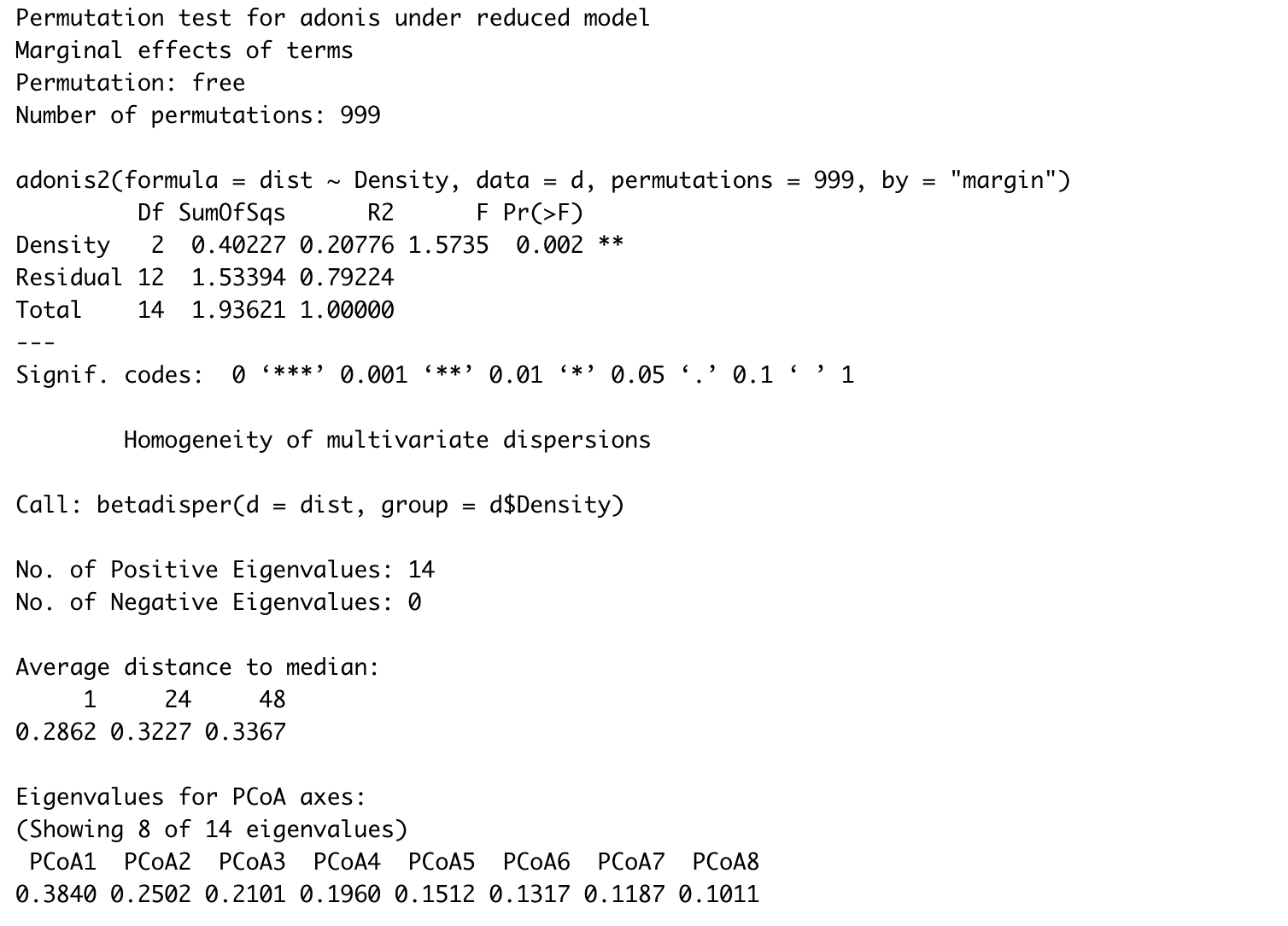


Alfalfa-Brassica Mixture


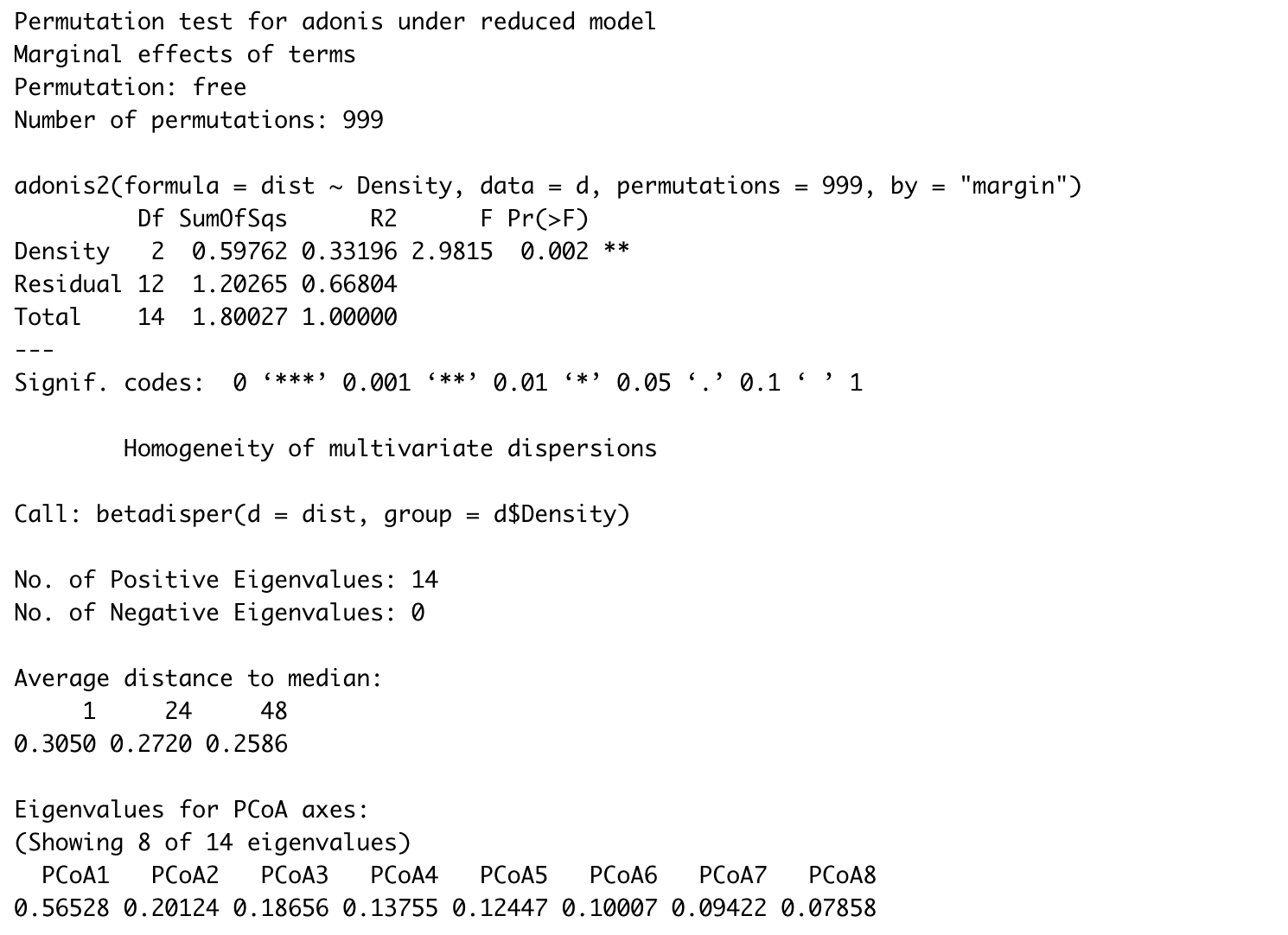


Alfalfa-Fescue Mixture


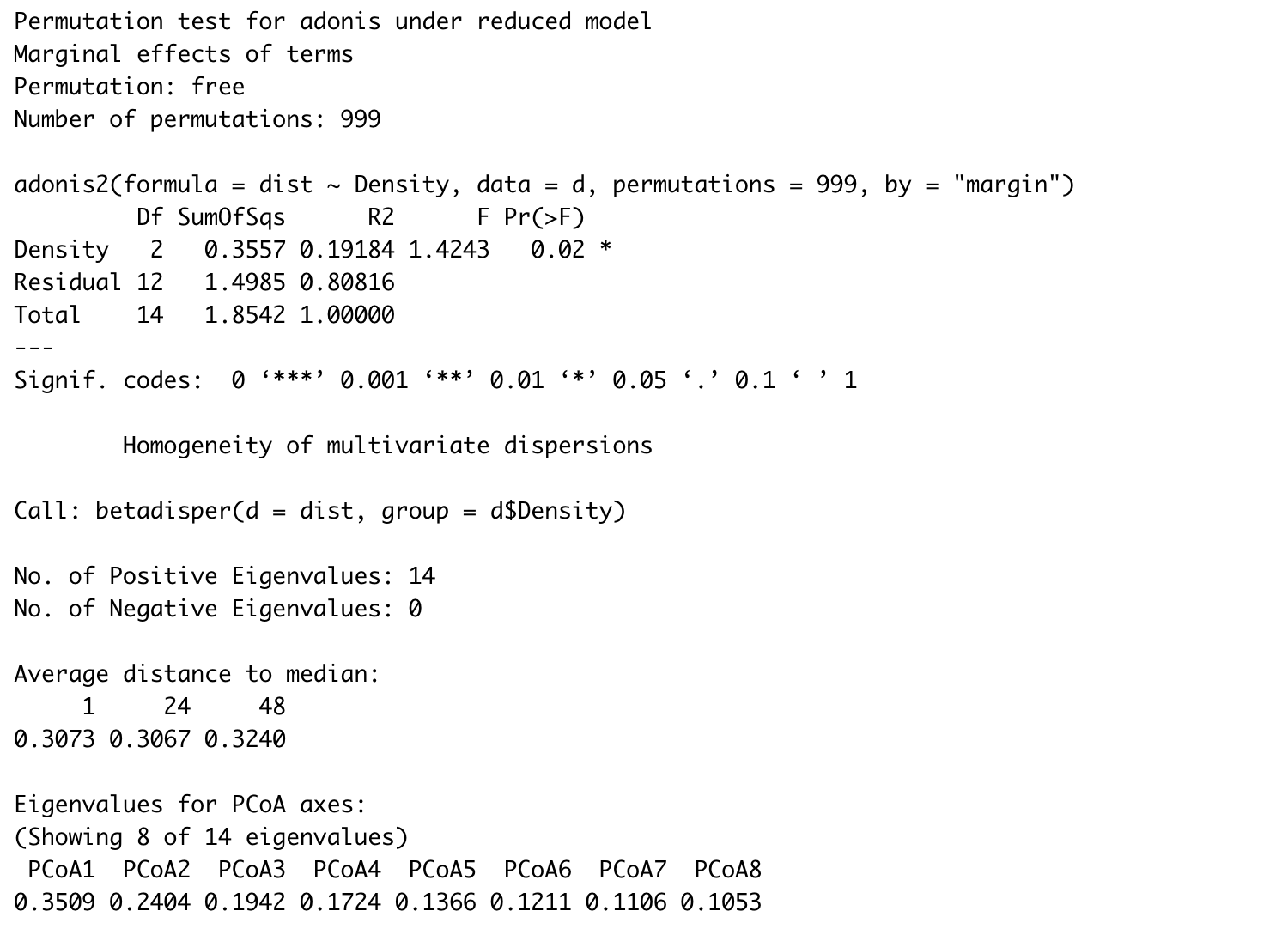


Brassica-Fescue Mixture


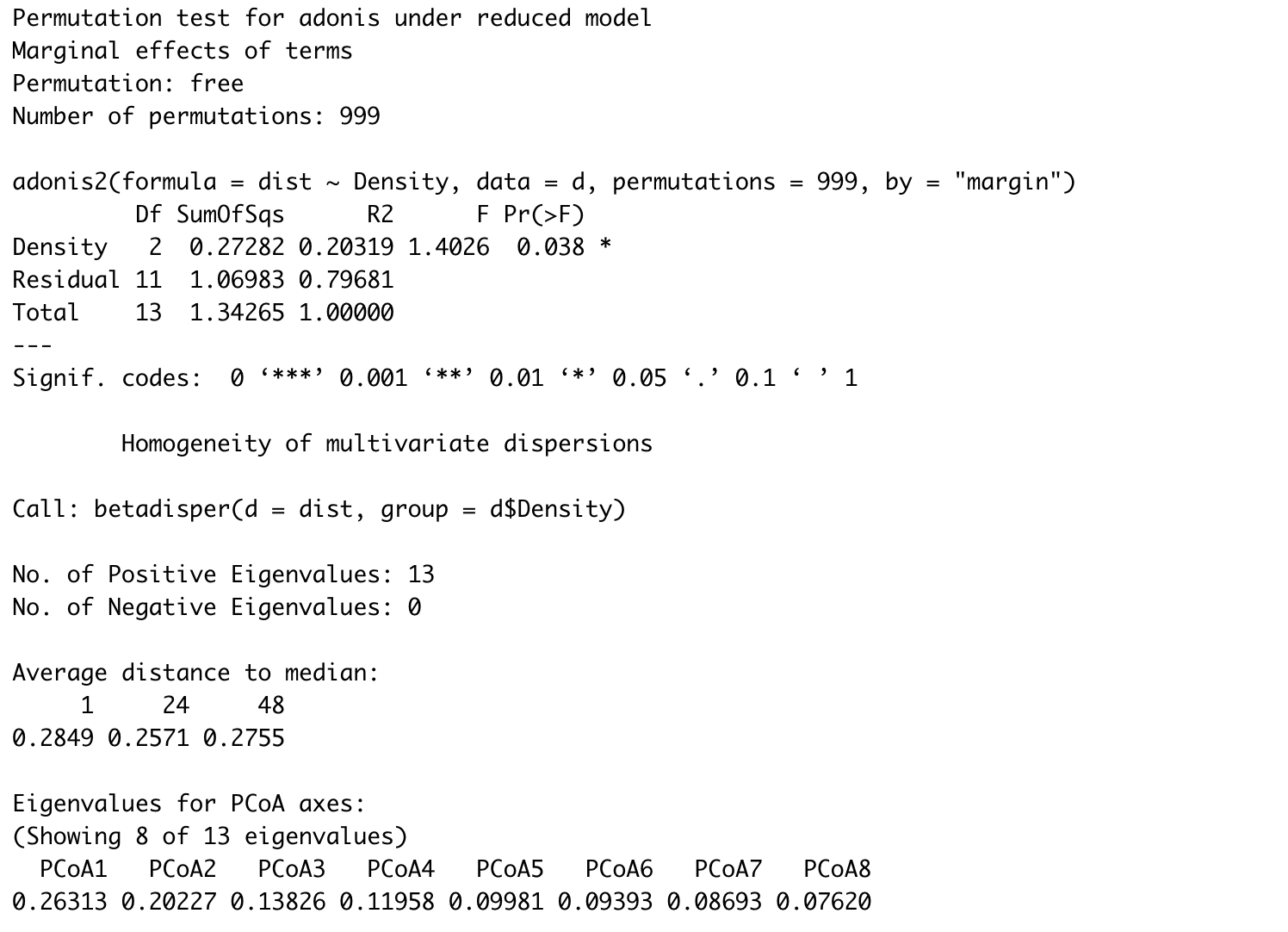


Alfalfa-Brassica-Fescue Mixture


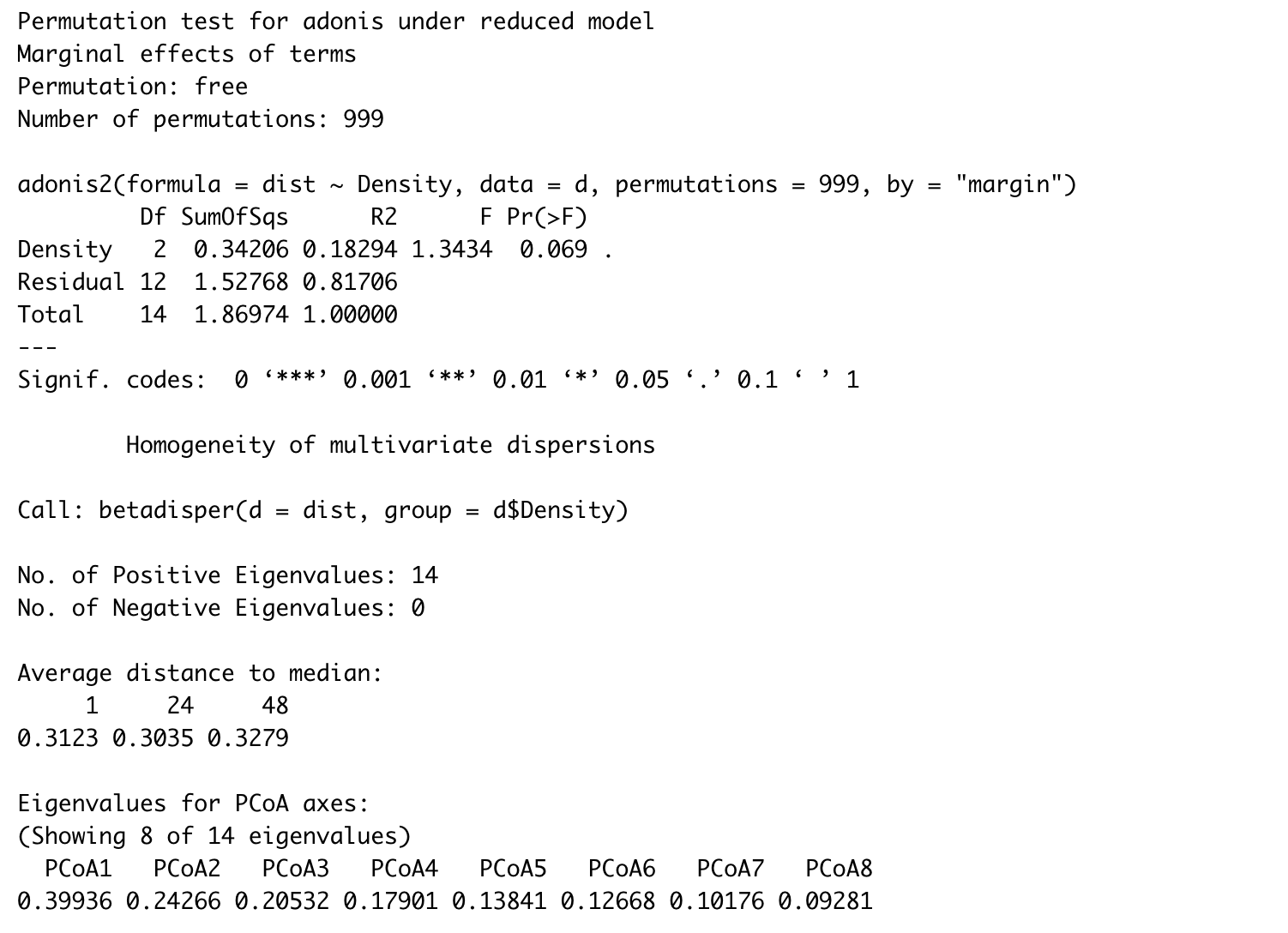


Supplementary Table S3: Aboveground plant biomass per individual plant by treatment

| Alfalfa Treatment | Individual Plant weight (g) | Brassica Treatment | Individual Plant weight (g) | Fescue Treatment | Individual Plant weight (g) |
| --- | --- | --- | --- | --- | --- |
| **Alfalfa** 1 | 0.349 | **Brassica** 1 | 0.344 | **Fescue** 1 | 0.168 |
| **Alfalfa-**brassica1 | 0.109 | alfalfa-**Brassica** 1 | 0.316 | alfalfa-**Fescue** 1 | 0.125 |
| **Alfalfa**-fescue 1 | 0.334 | **Brassica-**fescue 1 | 0.311 | brassica-**Fescue** 1 | 0.011 |
| **Alfalfa**-brassica-fescue 1 | 0.097 | alfalfa-**Brassica**-fescue 1 | 0.249 | alfalfa-brassica-**Fescue** 1 | 0.016 |
| **Alfalfa** 24 | 0.037 | **Brassica** 24 | 0.016 | **Fescue** 24 | 0.014 |
| **Alfalfa-**brassica 24 | 0.018 | alfalfa-**Brassica** 24 | 0.023 | alfalfa-**Fescue** 24 | 0.018 |
| **Alfalfa**-fescue 24 | 0.048 | **Brassica-**fescue 24 | 0.026 | brassica-**Fescue** 24 | 0.004 |
| **Alfalfa**-brassica-fescue 24 | 0.022 | alfalfa-**Brassica**-fescue 24 | 0.028 | alfalfa-brassica-**Fescue** 24 | 0.004 |
| **Alfalfa** 48 | 0.031 | **Brassica** 48 | 0.012 | **Fescue** 48 | 0.010 |
| **Alfalfa-**brassica 48 | 0.016 | alfalfa-**Brassica** 48 | 0.013 | alfalfa-**Fescue** 48 | 0.010 |
| **Alfalfa**-fescue 48 | 0.041 | **Brassica-**fescue 48 | 0.012 | brassica-**Fescue** 48 | 0.004 |
| **Alfalfa**-brassica-fescue 48 | 0.016 | alfalfa-**Brassica**-fescue 48 | 0.013 | alfalfa-brassica-**Fescue** 48 | 0.004 |

Supplementary Table S4: Differential abundance results for alfalfa monocrop


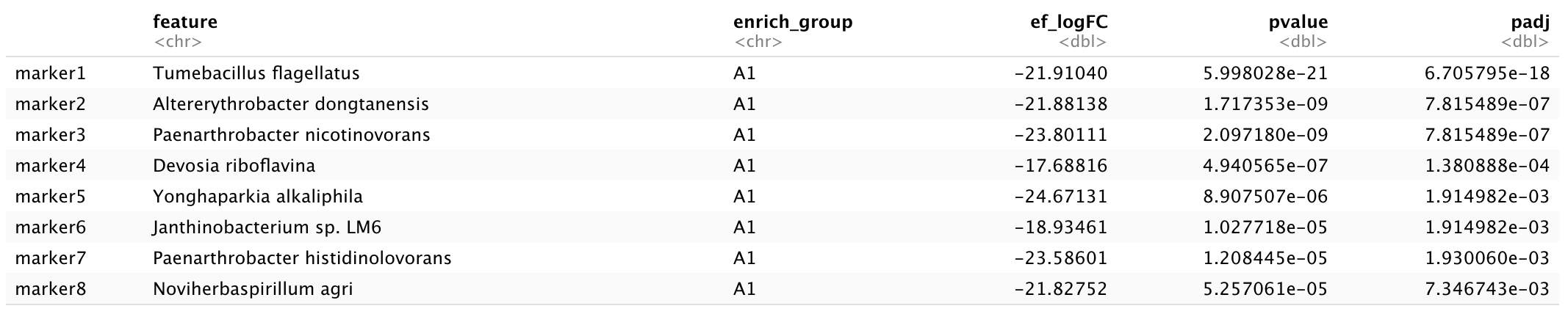


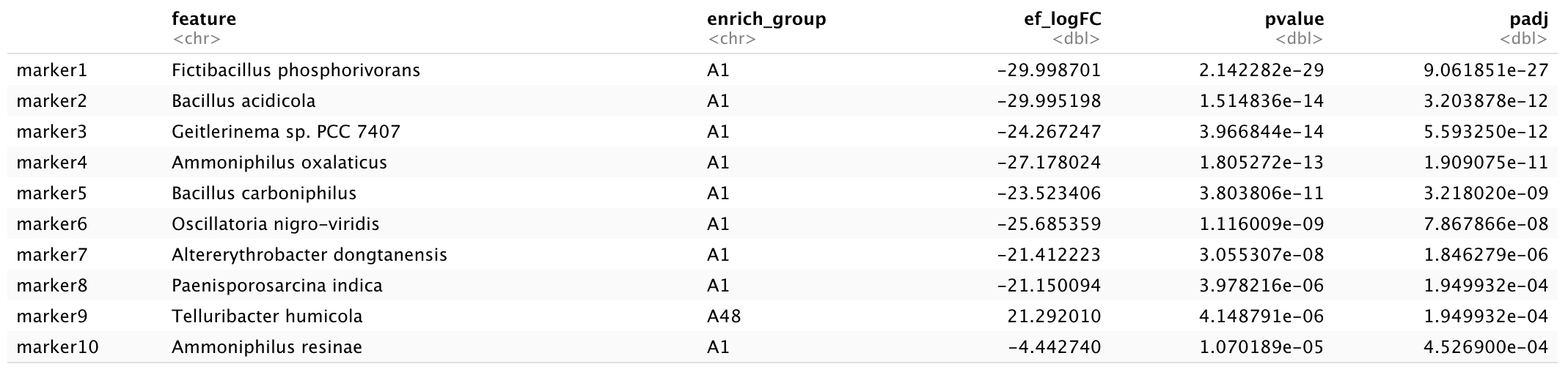

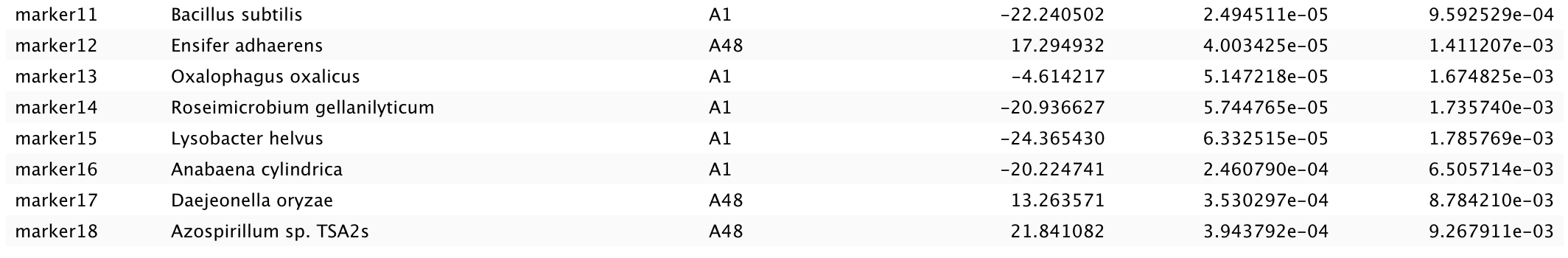


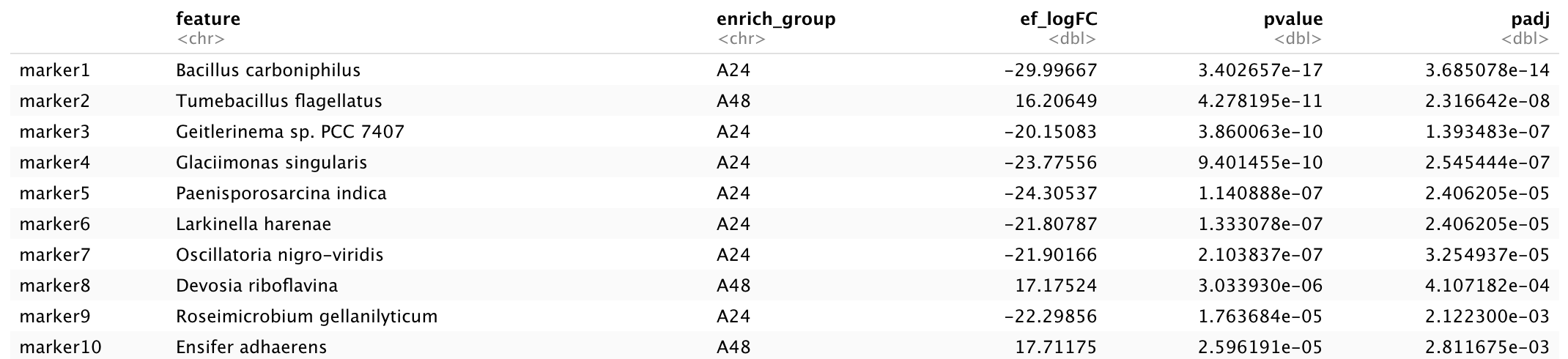


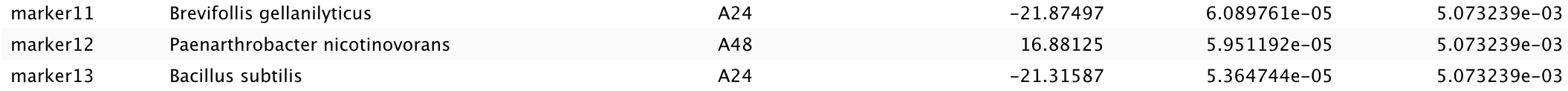


Supplementary Table S5: Differential abundance results for brassica monocrop


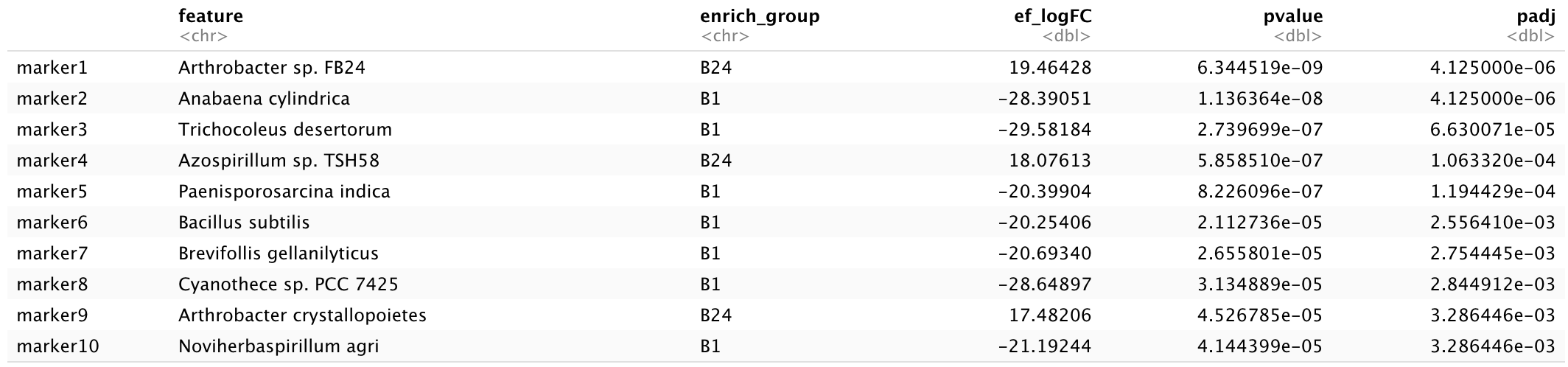


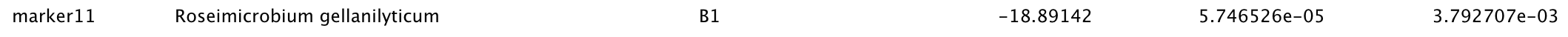


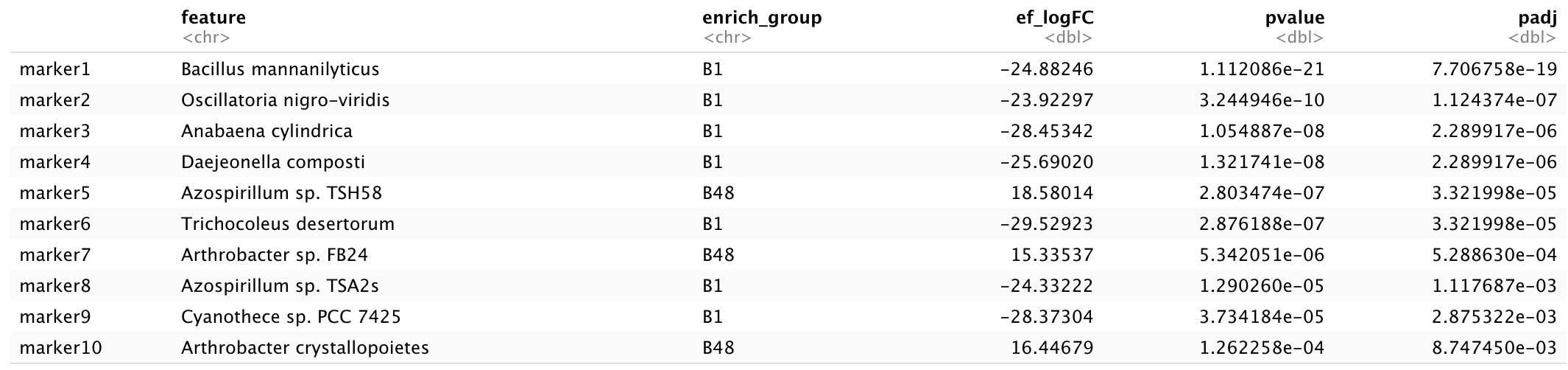


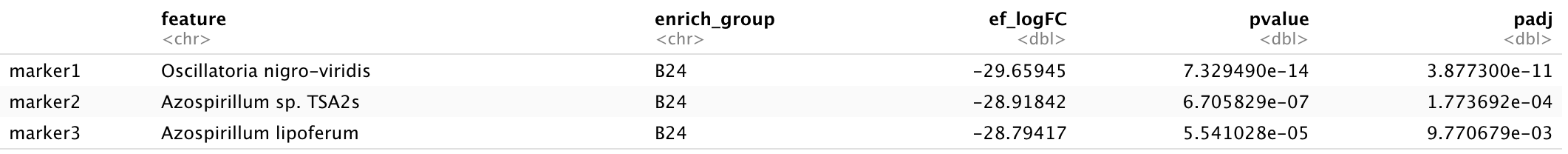


Supplementary Table S6: Differential abundance results for fescue monocrop


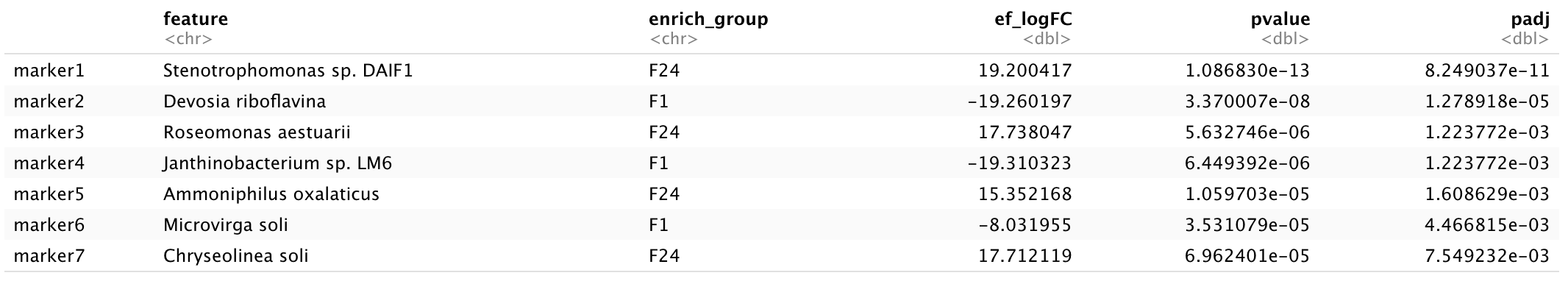


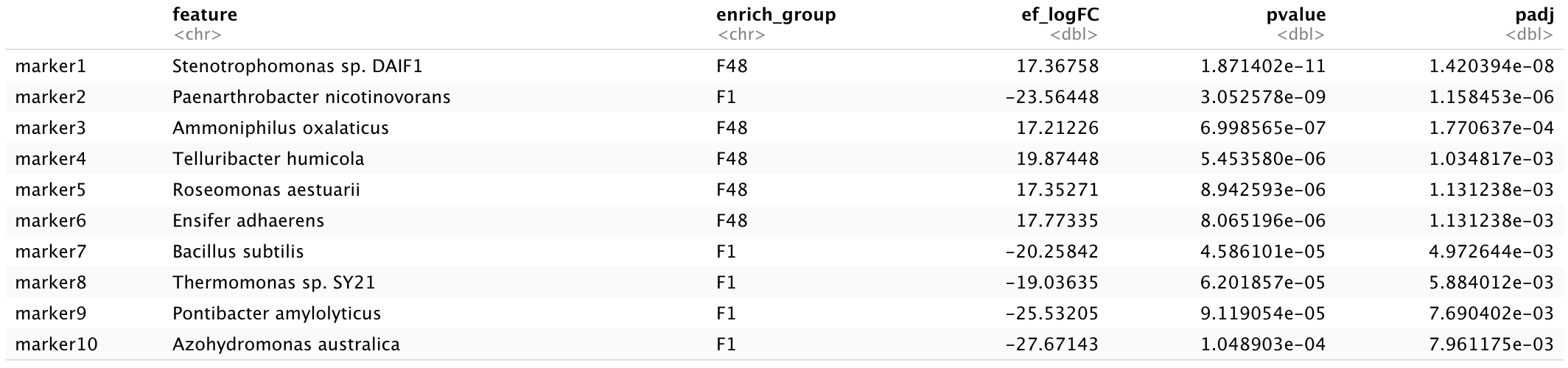


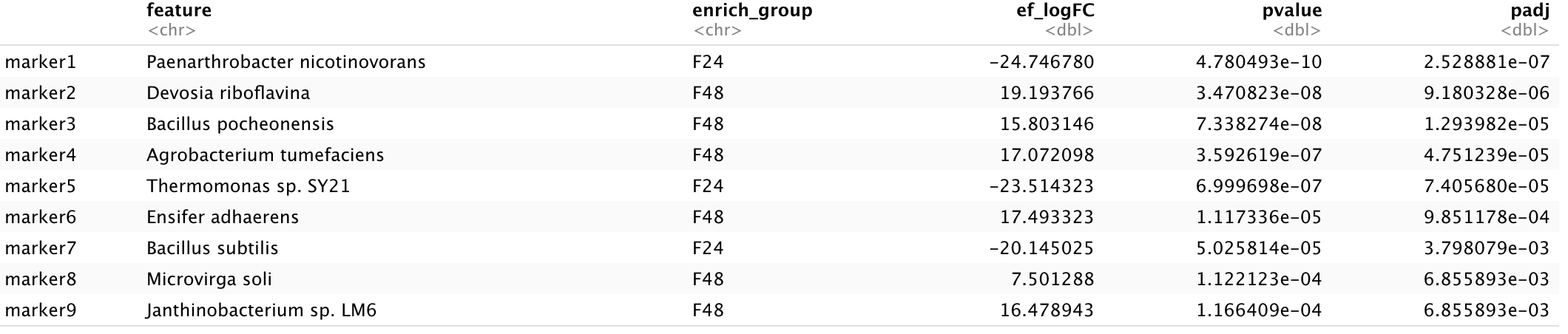


Supplementary Table S7: Differential abundance results for alfalfa-brassica mixture


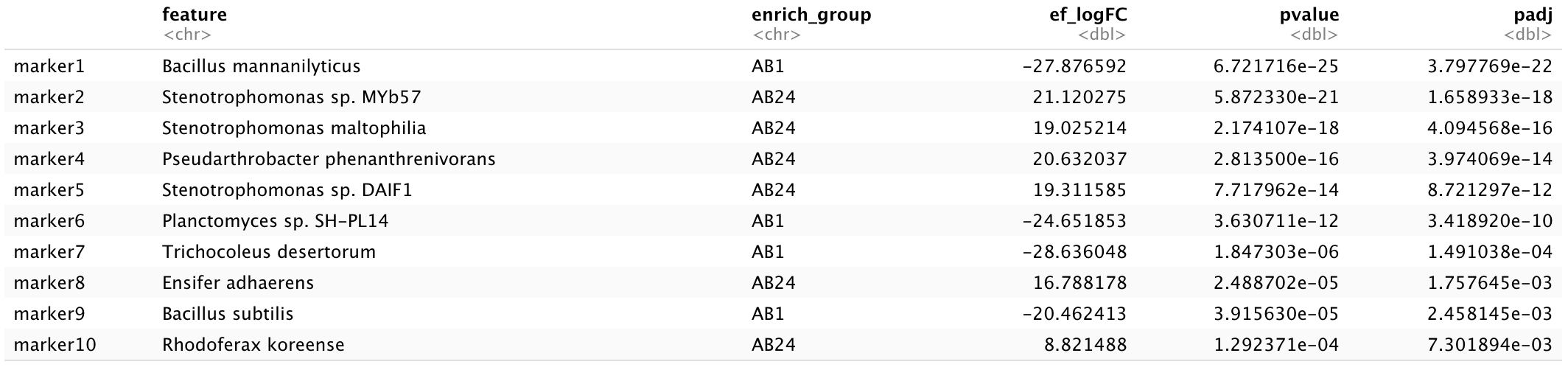


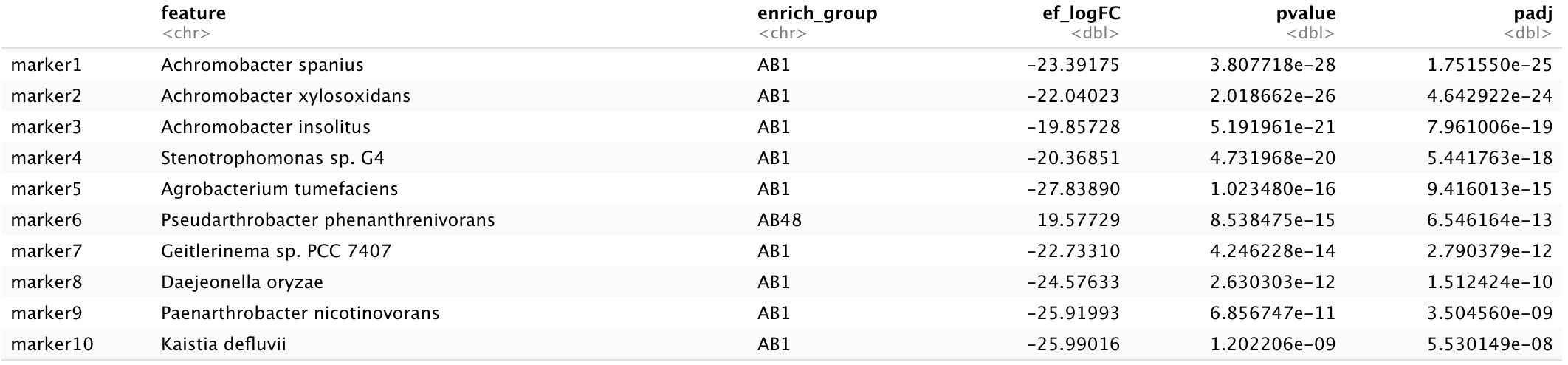


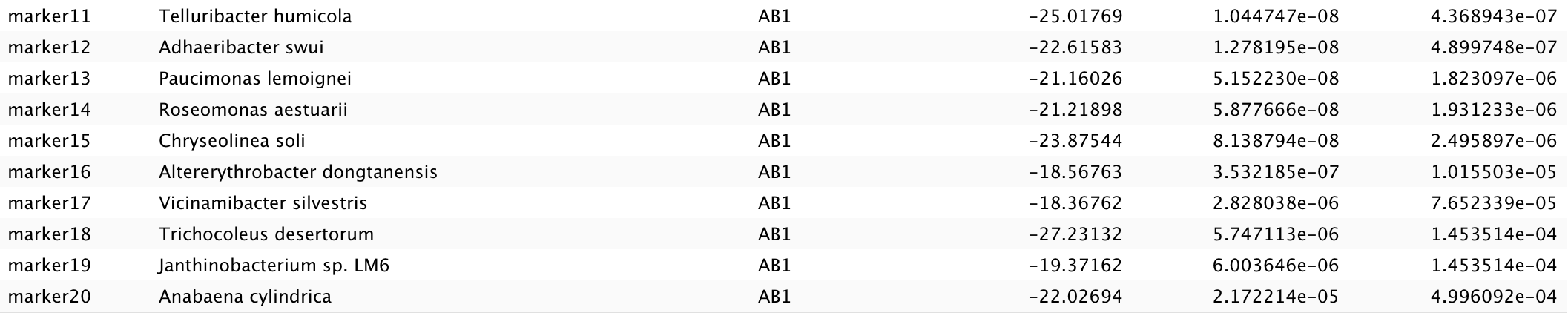


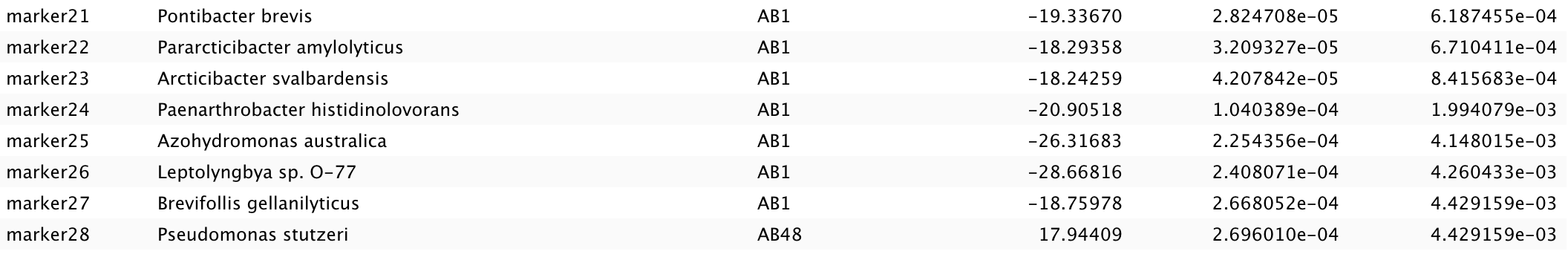


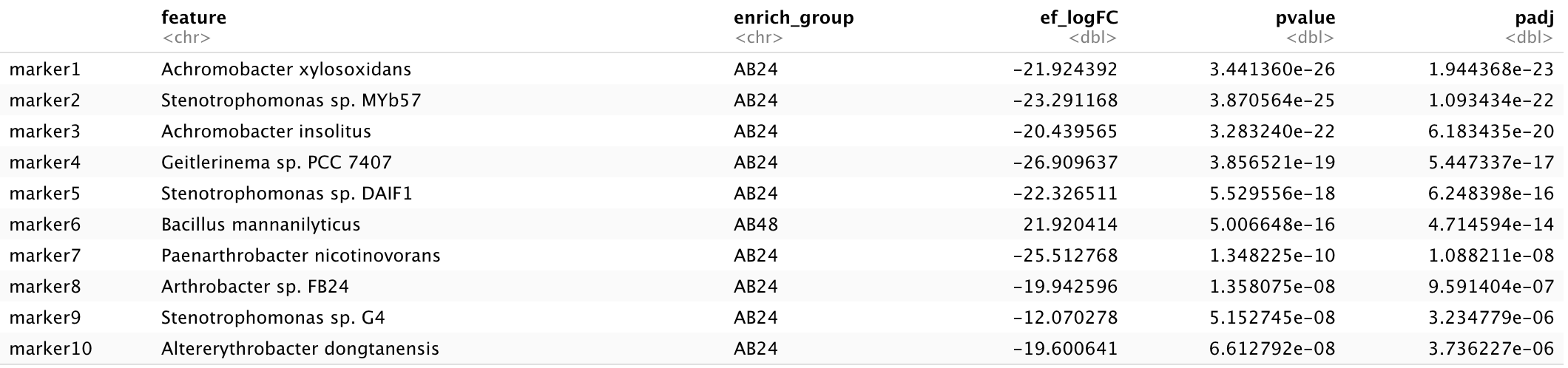


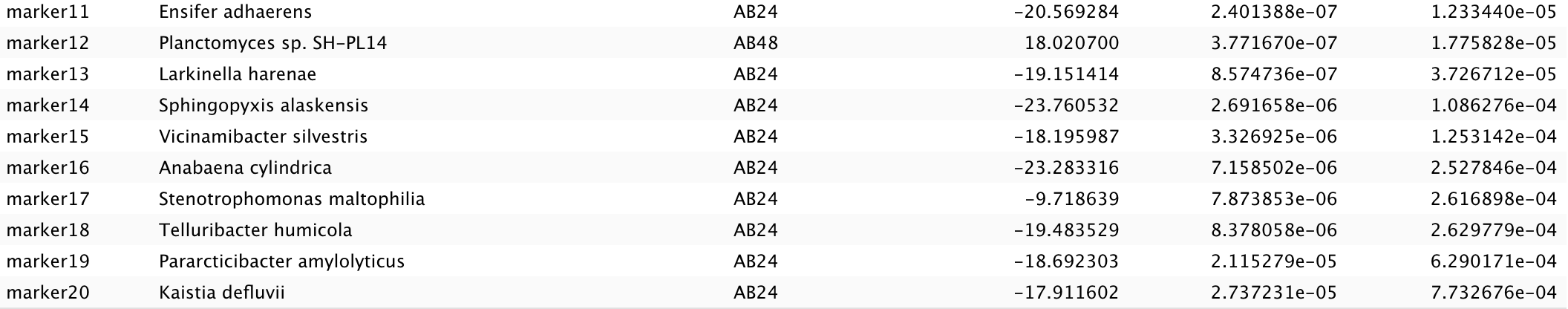


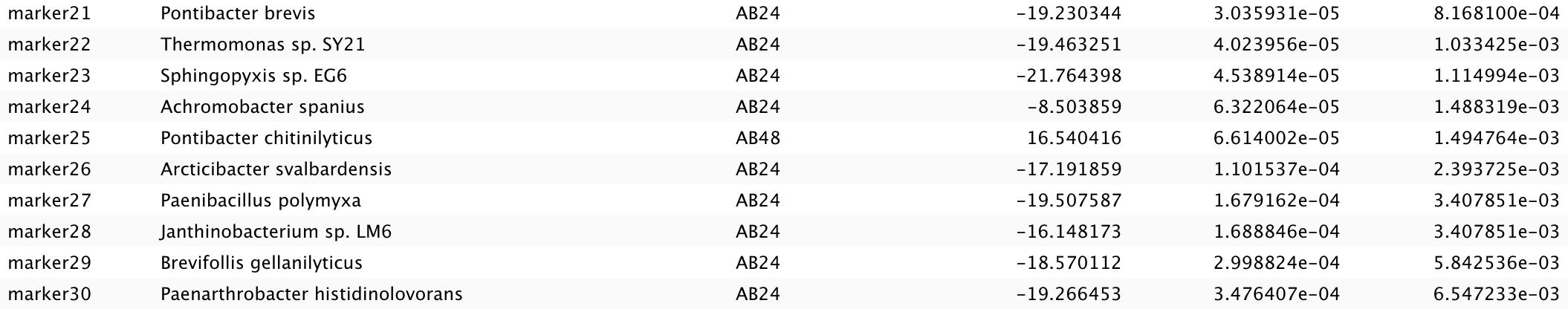


Supplementary Table S8: Differential abundance results for alfalfa-fescue mixture


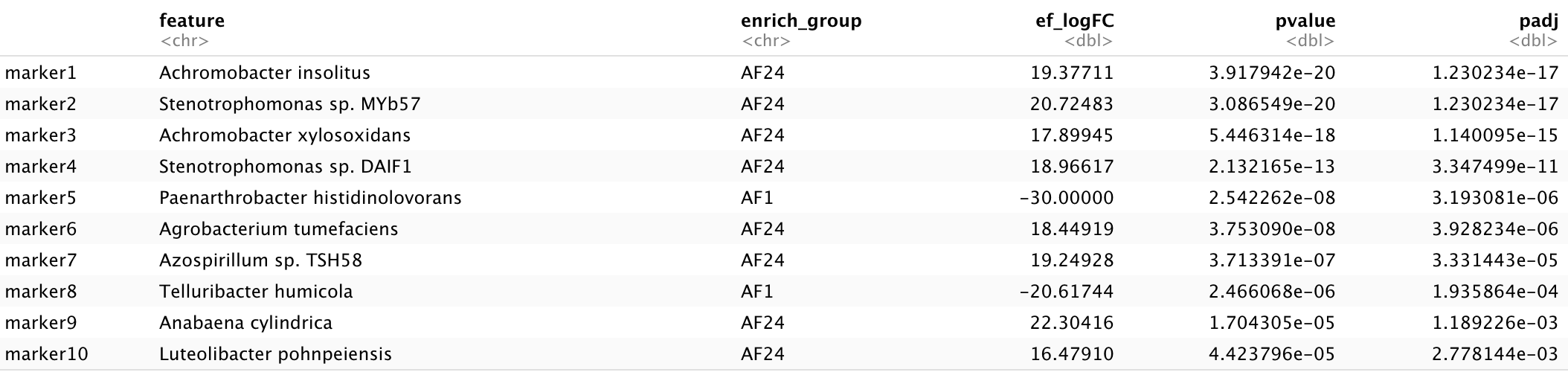


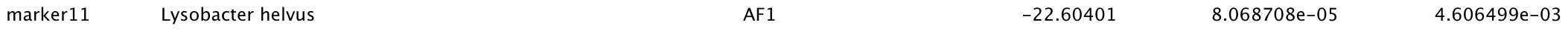


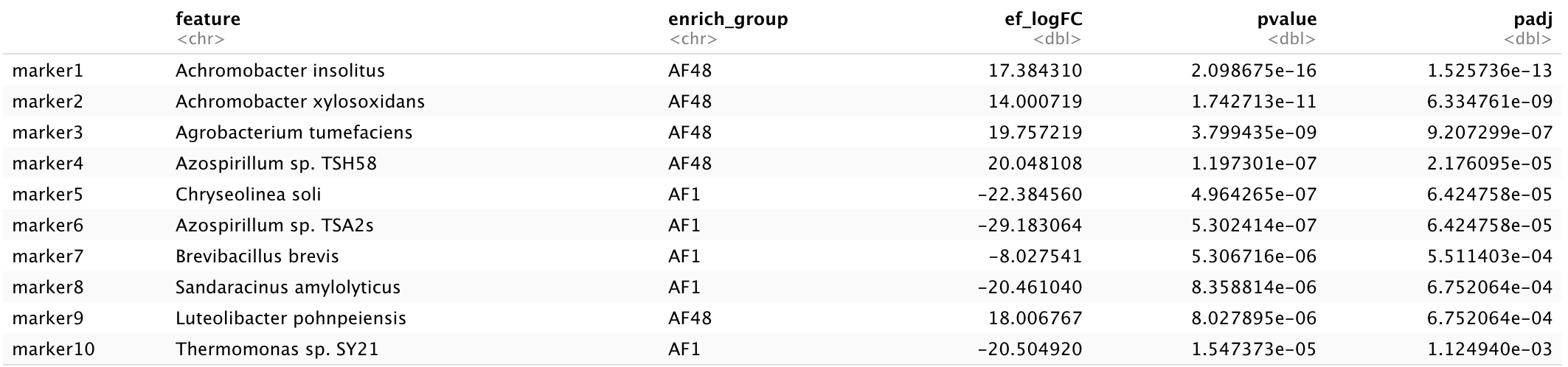


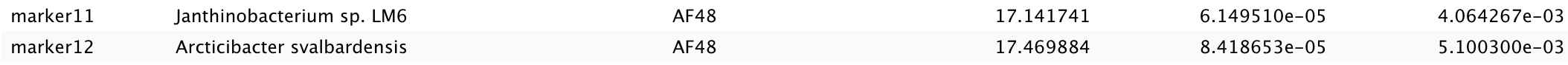


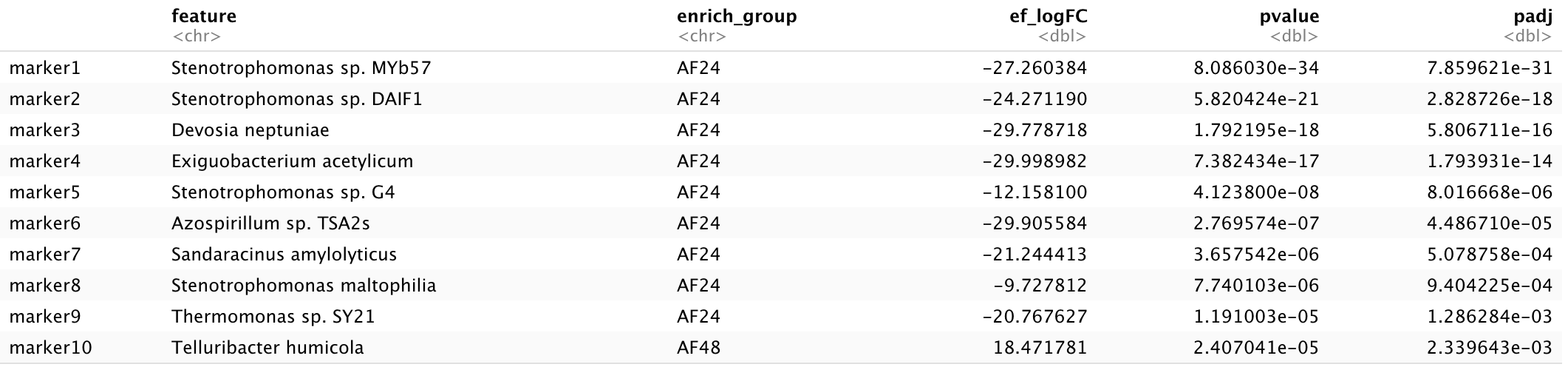


Supplementary Table S9: Differential abundance results for brassica-fescue mixture
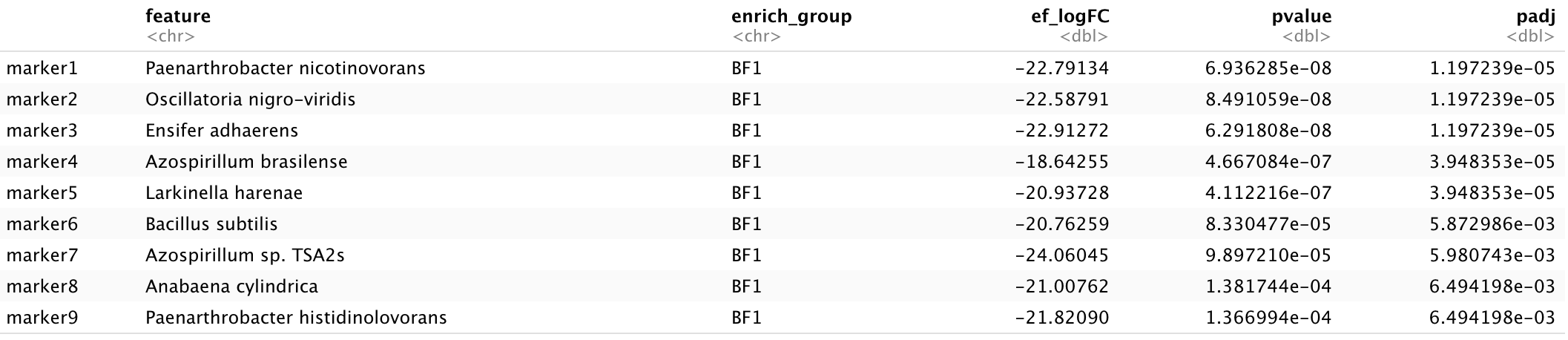


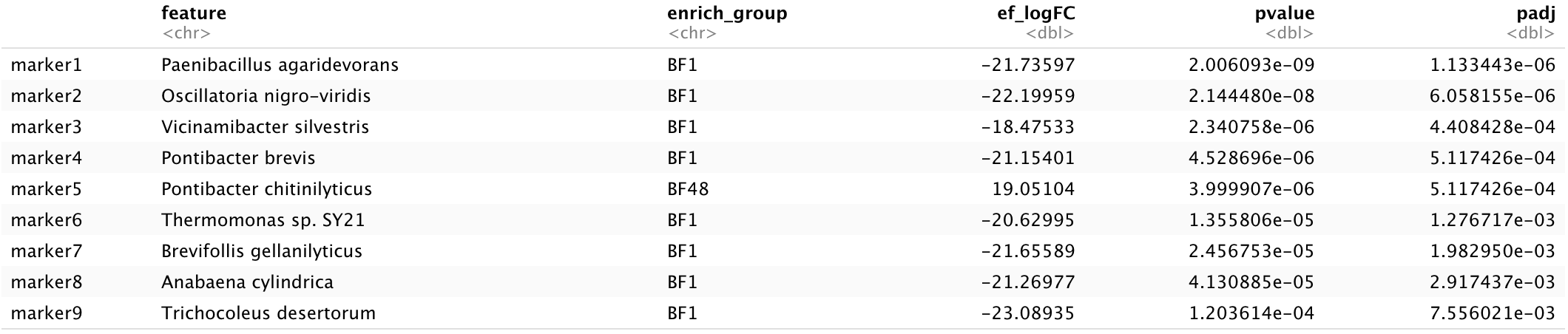


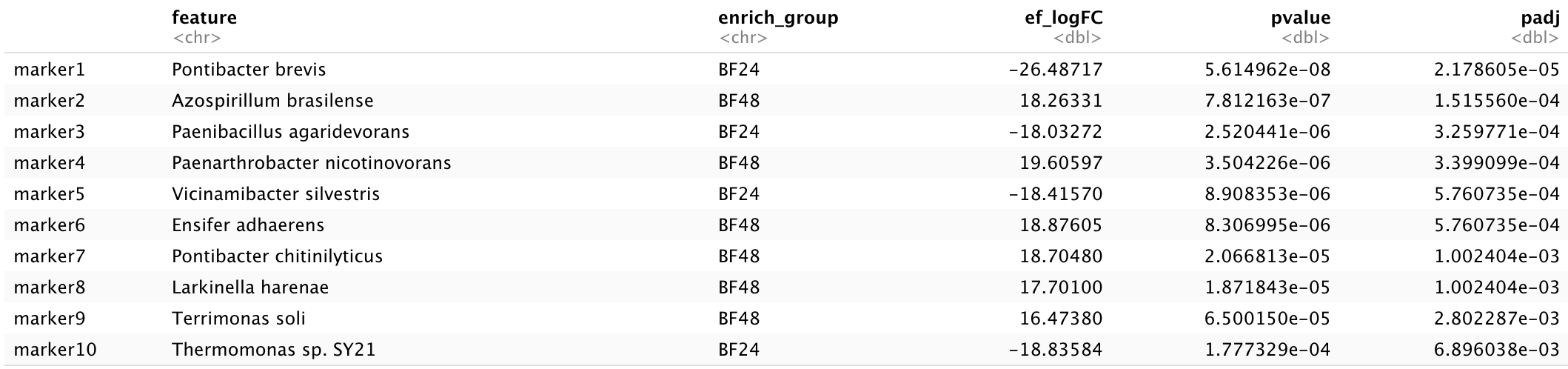


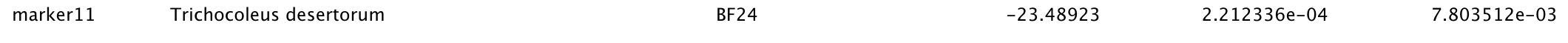


Supplementary Table S10: Differential abundance results for alfalfa-brassica-fescue mixture


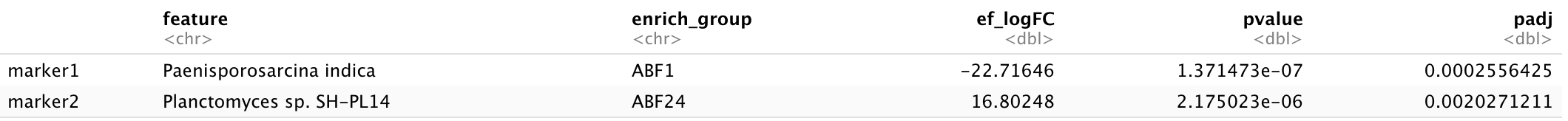


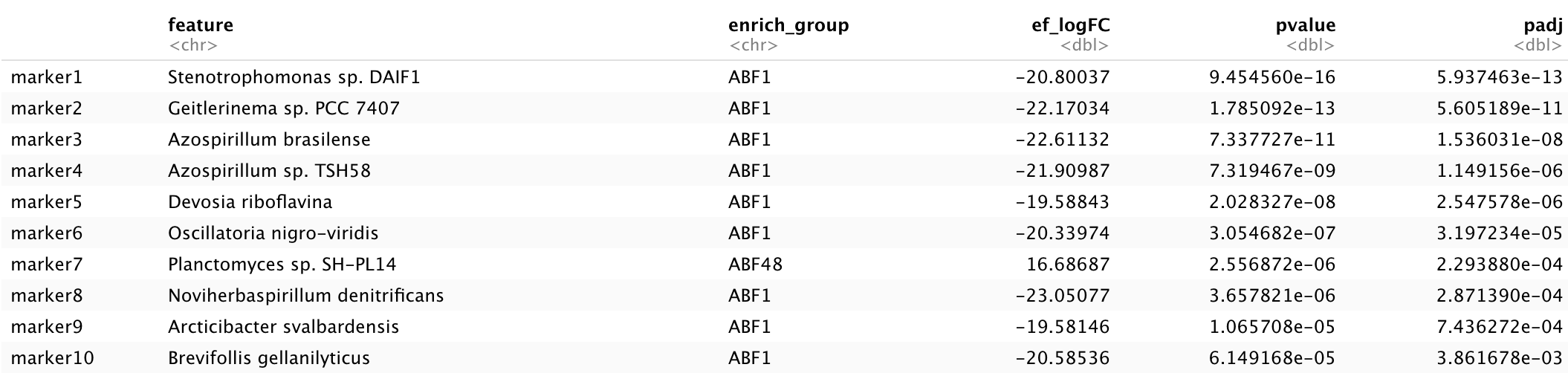


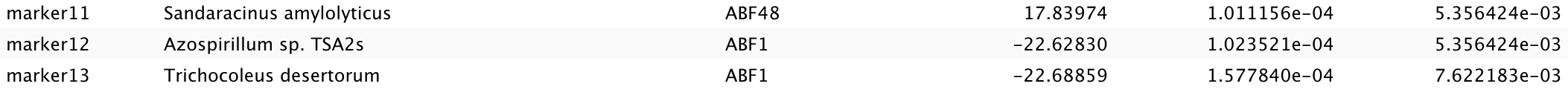


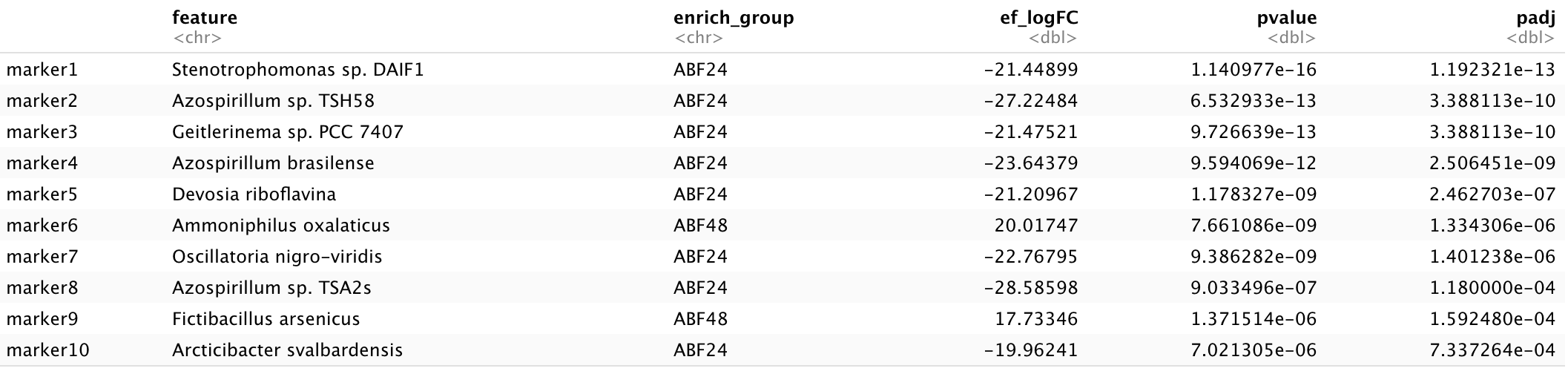


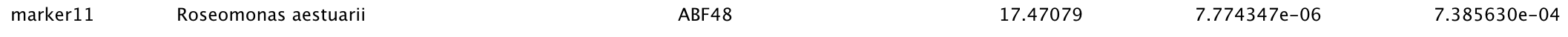


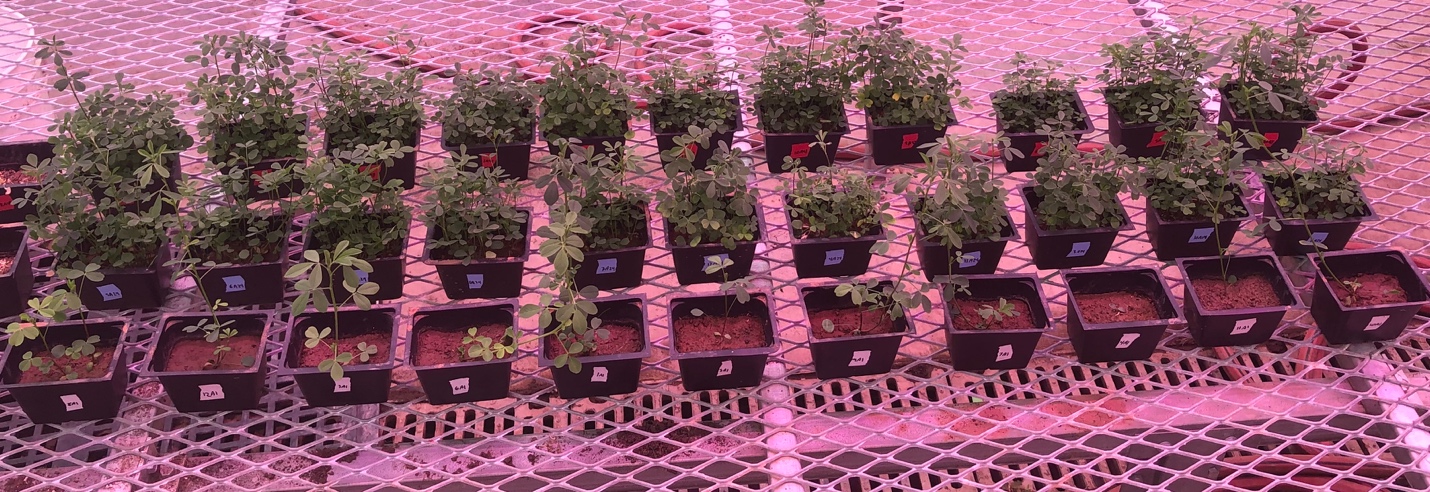


Supplementary Figure S6: Picture of the monocrop of alfalfa at increasing density (bottom row one plant, middle row 24 plants, and top row 48 plants).


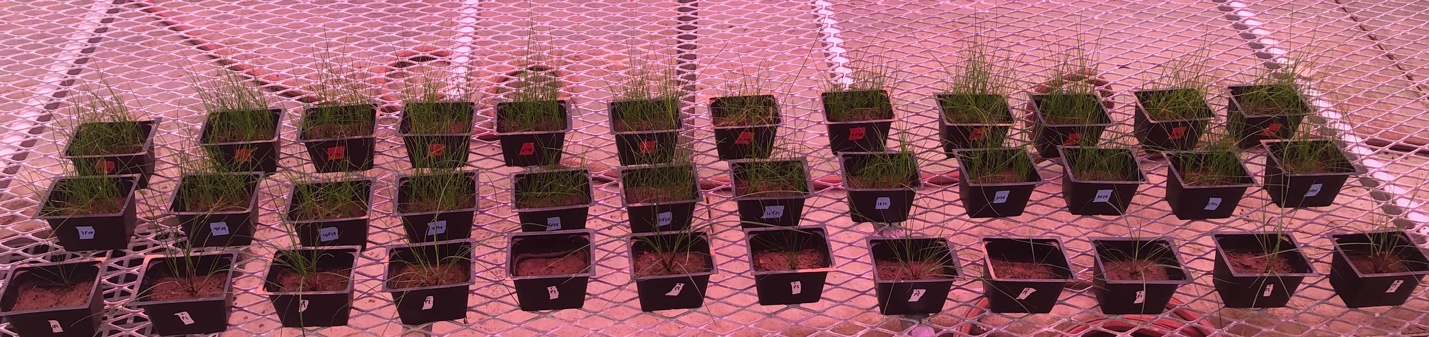


Supplementary Figure S7: Picture of the monocrop of fescue at increasing density (bottom row one plant, middle row 24 plants, and top row 48 plants).


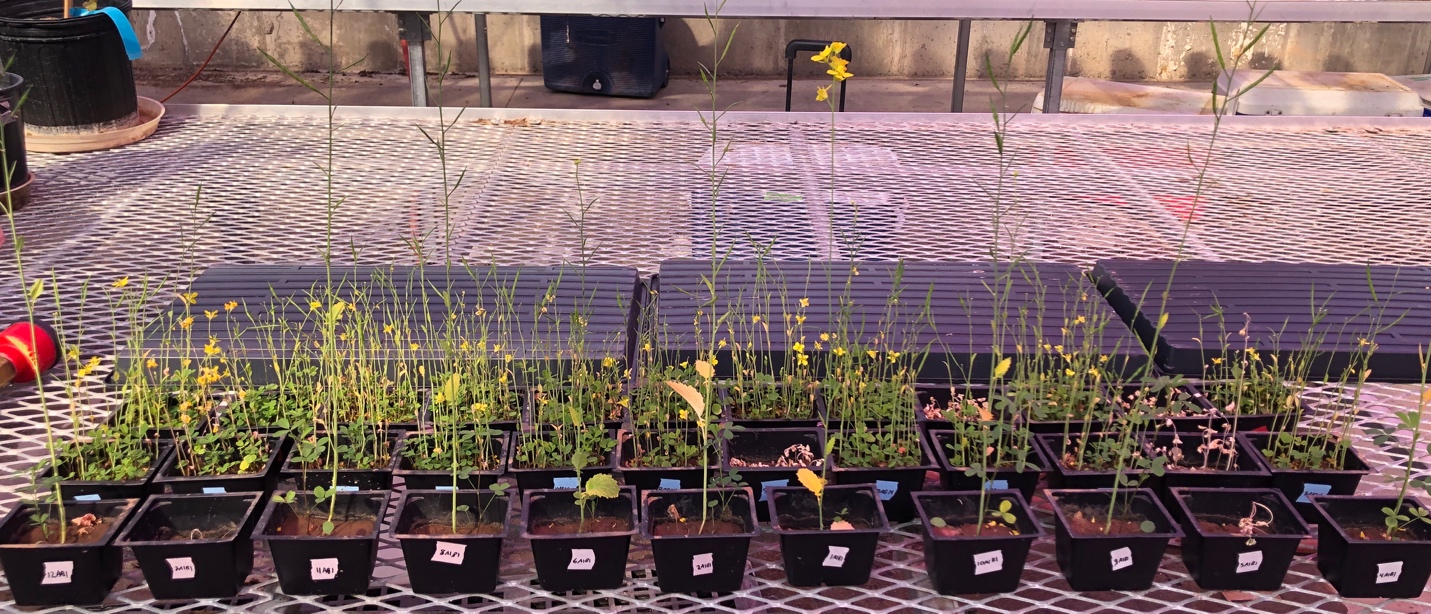


Supplementary figure S8: Supplementary figure 5: Picture of the alfalfa-brassica mixture at increasing density (bottom row one plant, middle row 24 plants, and top row 48 plants).
